# Ubiquitinome Profiling Reveals *in Vivo* UBE2D3 Targets and Implicates UBE2D3 in Protein Quality Control

**DOI:** 10.1016/j.mcpro.2023.100548

**Published:** 2023-04-13

**Authors:** Zeliha Yalçin, Daniëlle Koot, Karel Bezstarosti, Daniel Salas-Lloret, Onno B. Bleijerveld, Vera Boersma, Mattia Falcone, Román González-Prieto, Maarten Altelaar, Jeroen A.A. Demmers, Jacqueline J.L. Jacobs

**Affiliations:** 1 Division of Oncogenomics, The Netherlands Cancer Institute, Amsterdam, The Netherlands; 2 Proteomics Center, Erasmus Medical Center, Rotterdam, The Netherlands; 3 Department of Cell and Chemical Biology, Leiden University Medical Center, Leiden, The Netherlands; 4 Proteomics Facility, The Netherlands Cancer Institute, Amsterdam, The Netherlands; 5 Genome Proteomics Laboratory, Andalusian Center for Molecular Biology and Regenerative Medicine (CABIMER), University of Seville, Seville, Spain; 6 Department of Cell Biology, University of Seville, Seville, Spain; 7 Biomolecular Mass Spectrometry and Proteomics, Bijvoet Center for Biomolecular Research and Utrecht Institute for Pharmaceutical Sciences, Netherlands Proteomics Center, University of Utrecht, Utrecht, The Netherlands

**Keywords:** UBE2D3, diGly-proteomics, PQC, RPS10, SILAC

## Abstract

Ubiquitination has crucial roles in many cellular processes, and dysregulation of ubiquitin machinery enzymes can result in various forms of pathogenesis. Cells only have a limited set of ubiquitin-conjugating (E2) enzymes to support the ubiquitination of many cellular targets. As individual E2 enzymes have many different substrates and interactions between E2 enzymes and their substrates can be transient, it is challenging to define all *in vivo* substrates of an individual E2 and the cellular processes it affects. Particularly challenging in this respect is UBE2D3, an E2 enzyme with promiscuous activity *in vitro* but less defined roles *in vivo*. Here, we set out to identify *in vivo* targets of UBE2D3 by using stable isotope labeling by amino acids in cell culture–based and label-free quantitative ubiquitin diGly proteomics to study global proteome and ubiquitinome changes associated with UBE2D3 depletion. UBE2D3 depletion changed the global proteome, with the levels of proteins from metabolic pathways, in particular retinol metabolism, being the most affected. However, the impact of UBE2D3 depletion on the ubiquitinome was much more prominent. Interestingly, molecular pathways related to mRNA translation were the most affected. Indeed, we find that ubiquitination of the ribosomal proteins RPS10 and RPS20, critical for ribosome-associated protein quality control, is dependent on UBE2D3. We show by Targets of Ubiquitin Ligases Identified by Proteomics 2 methodology that RPS10 and RPS20 are direct targets of UBE2D3 and demonstrate that the catalytic activity of UBE2D3 is required to ubiquitinate RPS10 *in vivo*. In addition, our data suggest that UBE2D3 acts at multiple levels in autophagic protein quality control. Collectively, our findings show that depletion of an E2 enzyme in combination with quantitative diGly-based ubiquitinome profiling is a powerful tool to identify new *in vivo* E2 substrates, as we have done here for UBE2D3. Our work provides an important resource for further studies on the *in vivo* functions of UBE2D3.

## Highlights

• SILAC-based and label-free diGly proteomics to identify *in vivo* substrates of UBE2D3.
• UBE2D3 affects CRABP1 and TSPAN8 protein levels.
• UBE2D3 regulates ubiquitination of proteins involved in protein quality control.
• UBE2D3 ubiquitinates RPS10 and RPS20 *in vivo*.

## Graphical Abstract

**Figure:**
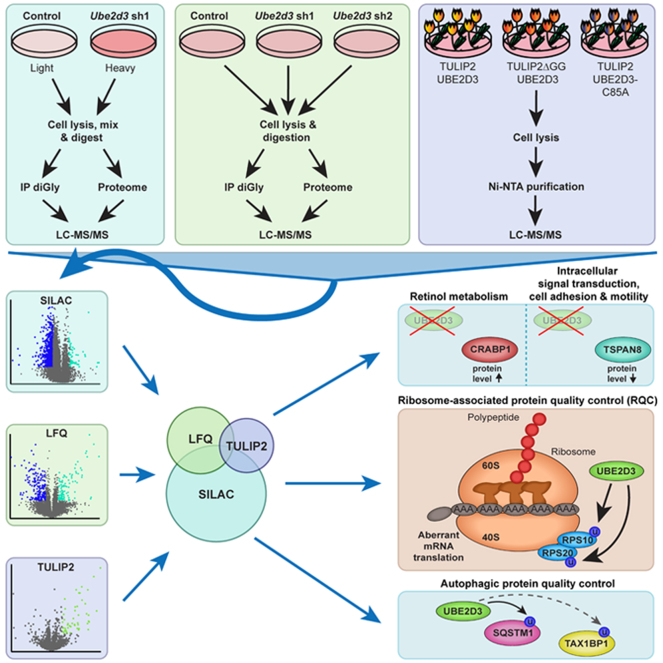

## In Brief

To identify *in vivo* substrates of the E2 ubiquitin-conjugating enzyme UBE2D3, we combined SILAC-based and label-free diGly-proteomics with UBE2D3 depletion. We show that UBE2D3 impacts CRABP1 and TSPAN8 levels and ubiquitinates key proteins in protein quality control. TULIP2 methodology identifies RPS10, RPS20, and SQSTM1 among direct targets of UBE2D3, and we show that UBE2D3's catalytic activity is required for RPS10 ubiquitination *in vivo*. Our approach identifies many potential UBE2D3 substrates, thereby providing a resource for future studies on UBE2D3 functions.

Ubiquitination is a post-translational modification in which the conserved 76 amino-acid protein ubiquitin is attached to a lysine residue of a target protein by the combined actions of ubiquitin-activating (E1) enzymes, ubiquitin-conjugating (E2) enzymes, and ubiquitin (E3) ligases. This mostly occurs through the formation of an isopeptide bond between the C-terminal glycine carboxyl moiety of ubiquitin and the ε-amino group of a lysine residue on the substrate protein, but ubiquitin can also be attached to the amino terminus of substrates. Deubiquitinating enzymes (DUBs) remove ubiquitin from targets, to maintain cellular ubiquitin homeostasis (1, 2). Ubiquitination of a target protein can have various consequences, such as proteasomal degradation or changes in protein activity, but it can also act in signal transduction or affect the cellular localization of the target protein. Proteins can be monoubiquitinated, in which one ubiquitin molecule is attached to one lysine residue of the target, or multimonoubiquitinated, in which the target is modified with one ubiquitin molecule at multiple lysines. In addition, proteins can be polyubiquitinated, in which a ubiquitin chain is formed on the initially attached ubiquitin. The linkage type can determine the consequence of the ubiquitination. For example, K48-linked ubiquitin chains are known to promote proteasomal degradation of the ubiquitinated protein, whereas K63-linked ubiquitin chains mainly play a role in signaling events (1, 3). Mammalian cells contain two E1 enzymes, ±40 E2 enzymes, >600 E3 enzymes, and ±100 DUBs (2, 3). Together, these enzymes need to coordinate all ubiquitination reactions in the cell and therefore have many substrates each. A major challenge is to identify all substrates of individual enzymes. Not only because of the number of substrates, but also because interactions between these enzymes and their substrates are often very transient and therefore difficult to detect. Identifying as many targets of ubiquitin-system enzymes as possible increases knowledge on the cellular processes affected by the ubiquitin system, helps predicting possible secondary or adverse effects of targeting these enzymes, and potentially helps the development of more specific drugs for treatment of pathologies.

In this study, we focus on the discovery of new *in vivo* targets of UBE2D3 (also known as UBCH5C), a member of the UBE2D (or UBCH5) family of E2 enzymes (4). *In vitro* UBE2D3 is a very promiscuous E2 enzyme. It is frequently used in *in vitro* ubiquitination assays because it is often the most active E2 enzyme in such assays, functioning with almost every E3 ligase in ubiquitination of target proteins (5, 6, 7, 8). Therefore, many *in vitro* substrates and interacting E3 ligases have been identified for this E2. However, *in vivo* ubiquitination is highly controlled, and much less is known about the *in vivo* targets and E3 enzymes that UBE2D3 partners with to ubiquitinate these target proteins. Nevertheless, UBE2D3 has been linked to multiple important cellular processes *in vivo*. For instance, UBE2D3 contributes to keeping p53 tumor suppressor levels low in unstressed cells by acting with the E3 ligase MDM2 to ubiquitinate p53 and thereby target it for proteasomal degradation (9). Also, UBE2D3 contributes to early stages of DNA repair by the homologous recombination repair machinery by acting with RNF138 to promote ubiquitination and accrual of the DNA end-resection promoting factor CtIP (10). Moreover, aberrant UBE2D3 activity is associated with pathology. *UBE2D3* is mutated, and its expression level is altered in a wide variety of cancers, including breast, ovarian, cervical, head and neck and esophageal cancer, melanoma, leukemia, and multiple myeloma (Oncomine, EMBL-EBI Expression Atlas, Cosmic, and ICGC databases). Furthermore, UBE2D3 levels affect responses to cancer cell treatment with radiation or all-*trans* retinoic acid (ATRA) (11, 12). To better understand the function of UBE2D3, the biological processes it affects, and the potential consequences of targeting this enzyme in a disease setting, it is relevant to expand knowledge on the direct and indirect *in vivo* targets of this E2.

In the past years, different proteomic approaches have been developed to characterize the ubiquitinome and identify substrates of E3 ligases (13, 14, 15). These are based on specific antibody-mediated enrichment of diGly peptides, which result from the trypsinization of ubiquitinated proteins (16, 17). Here, we combined ubiquitin diGly proteomics with stable isotope labeling of amino acids in cell culture (SILAC), allowing for quantitative identification of ubiquitinated proteins and mapping of modified lysines on the substrates (16, 17, 18, 19, 20). We integrated this with RNA interference–mediated depletion of the E2 UBE2D3 to identify new *in vivo* substrates for UBE2D3 and used a modified TULIP2 (Targets of Ubiquitin Ligases Identified by Proteomics 2) methodology to confirm substrates as direct targets of UBE2D3 (21). We show how UBE2D3 affects the proteome and, much more prominently, the ubiquitinome. Our analysis reveals important roles for UBE2D3 in metabolic pathways, cell adhesion, cell signaling, mRNA translation, and protein quality control (PQC). Most notably, we show that UBE2D3 regulates CRABP1 and TSPAN8 protein levels and that ubiquitination of the ribosomal proteins RPS10 and RPS20 by ZNF598, important for functional ribosome-associated PQC (RQC) (22, 23, 24), is dependent on UBE2D3 *in vivo*. We identify RPS10 and RPS20 as direct targets of UBE2D3 and show that the catalytic activity of UBE2D3 is indeed required for the ubiquitination of RPS10 *in vivo*.

Collectively, the quantitative ubiquitinome profiling in combination with UBE2D3 depletion that we performed here is a powerful source for insights into the spectrum of UBE2D3 substrates and the biological processes affected by UBE2D3, including its role in mRNA translation.

## Experimental Procedures

### Cell Culture

All cells were grown in Dulbecco's modified Eagle's medium with 100 U penicillin, 0.1 mg ml^−1^ streptomycin, 2 mM l-glutamine, and 10% fetal bovine serum. *Trf2*^*−/−*^; *p53*^*−/−*^; Trf2^Ile468Ala^ mouse embryonic fibroblasts (TRF2ts MEFs) were described before and maintained at 32 °C (25). This cellular model was chosen as in the context of other work we have been using this same p53-deficient cell line to study the potential impact of UBE2D3 in cells exposed to conditional TRF2 inactivation. This cell line allows for comparison between conditions of WT or perturbed TRF2 function. However in the work presented in this article, we do not make use of this property. HeLa, Phoenix, and human embryonic kidney 293T (HEK 293T) cells (American Type Culture Collection) were grown at 37 °C.

### Retroviral and Lentiviral Transductions

For the production of retrovirus, ecotropic phoenix producer cells were seeded on a 10 cm dish and transfected with 20 μg of retroviral vector DNA using CaPO_4_ precipitation. Medium was refreshed at 16 and 24 h after transfection, and viral supernatants were collected at approximately 48, 62, and 72 h post-transfection. Viral supernatants were either frozen in liquid nitrogen and stored at −80 °C until use or used immediately. For retroviral infection, cells were overlaid with viral supernatant supplemented with 4 μg ml^−1^ polybrene. Cells were infected a minimum of three times to achieve 100% transduction efficiency, which was confirmed by acquired resistance to selection drugs (4 μg ml^−1^ puromycin or 5 μg ml^−1^ blasticidin [Invitrogen]). Cells were transduced with pRetrosuper-puro or pRetrosuper-blast retroviruses encoding shRNA targeting mouse and human *UBE2D3* (*Ube2d3* sh1): 5′-TGGGTTTGGATCACATATC-3′, for which we confirmed by quantitative RT–PCR (qRT–PCR) that it specifically depletes *Ube2d3* and no other member of the UBE2D family. Control cells were transduced with empty pRetrosuper-puro or pRetrosuper-blast retrovirus. For the production of lentivirus, HEK 293T cells were transfected by CaPO_4_ precipitation with 10 μg of pLKO-puro shRNA lentiviral vectors obtained from Mission library clones (Sigma). Medium was refreshed at 16 h after adding the DNA–CaPO_4_ precipitate, and viral supernatants were collected at approximately 48 h post-transfection. Viral supernatants were either frozen in liquid nitrogen and stored at −80 °C until use or used immediately. For lentiviral infection, target cells were incubated for 16 h with viral supernatant that was supplemented with 4 μg ml^−1^ polybrene. Viral transduction was confirmed by acquired resistance to 2 μg ml^−1^ puromycin (Invitrogen). The following lentiviral shRNAs were used: human *UBE2D3* shRNA #1 TRCN0000038792: 5′-CCAGAGATTGCACGGATCTAT-3′, human *UBE2D3* shRNA #2 TRCN0000038790: 5′-GCCTGCTTTAACAATTTCTAA-3′, mouse *Ube2d3* shRNA #2 TRCN0000039469: 5′-ACAACAGAATATCTCGGGAAT-3′, and scrambled nontargeting shRNA: 5′-CAACAAGATGAAGAGCACCAA-3′. Human UBE2D3 WT and C85A mutant complementary DNA (cDNA) were cloned into a pCDH-puro backbone using traditional cloning and were used for lentiviral infections.

### SILAC Labeling

Control (pRetrosuper) or *Ube2d3* shRNA-transduced MEFs were grown in either light or heavy SILAC medium (SILAC Dulbecco's modified Eagle's medium, lysine(6) arginine(10) Kit; catalog no.: 282986434, Silantes), supplemented with 10% fetal bovine serum, 2 ml glutamine, 100 U penicillin, and 0.1 mg ml^−1^ streptomycin. Control MEFs were grown in light medium (Lys0, Arg0; ^12^C_6_ lysine and ^12^C_6_, ^14^N_4_ arginine). *Ube2d3* shRNA1-transduced MEFs were grown in heavy medium (Lys6, Arg10; ^13^C_6_ lysine and ^13^C_6_, ^15^N_4_ arginine). Cells were cultured for at least two weeks in SILAC light or heavy medium before the experiments were done to accomplish complete labeling. The SILAC experiments were performed and analyzed as six replicates.

### SILAC Sample Preparation

Cells grown in SILAC light or heavy medium were harvested for global proteome and ubiquitinome analyses. Cells were washed three times with ice-cold PBS and lysed in 200 μl of an 8 M urea/50 mM Tris–HCl (pH 8.0)/50 mM NaCl lysis buffer. Lysates were incubated on ice for 10 min and sonicated, debris was removed by centrifugation, and protein concentrations were determined using a bicinchoninic acid (BCA) assay (Pierce). Control and UBE2D3-depleted samples were mixed in a 1:1 ratio based on total protein content. An amount of 20 mg protein was used for diGly peptide enrichment for ubiquitinome analyses; for global proteome analyses, 0.5 mg lysate was used.

### Protein Digestion and Fractionation (SILAC)

Protein lysates were reduced with 10 mM DTT for 1 h at room temperature followed by alkylation with 55 mM chloroacetamide for 1 h in the dark. The mixture was diluted four times with 50 mM ammonium bicarbonate buffer before the addition of CaCl_2_ (1 mM final concentration). Proteins were digested with sequencing grade trypsin (1:100 [w:w]; Roche) overnight at room temperature. Alternatively, proteins were digested with LysC (1:100 [w:w]; Wako Chemicals) for 1 h at room temperature before trypsinization. Protein digests were then desalted using a Sep-Pak tC18 Vac cartridge (Waters) and eluted with 80% acetonitrile (AcN). Tryptic peptides were fractionated by hydrophilic interaction liquid chromatography on an Agilent 1100 HPLC system using a 5 μm particle size 4.6 × 250 mm TSKgel amide-80 column (Tosoh Biosciences). An amount of 200 μg of tryptic digest in 80% AcN was loaded onto the column. Peptides were eluted using a nonlinear gradient from 80% B (100% AcN) to 100% A (20 mM ammonium formate in water) with a flow of 1 ml/min. A total of 16 6 ml fractions were collected, lyophilized, and pooled into eight final fractions. Each fraction was then analyzed by nanoflow LC–MS/MS.

### DiGly Peptide Enrichment (SILAC)

DiGly-modified peptides were enriched by immunoprecipitation using PTMScan ubiquitin remnant motif (K-Ɛ-GG) antibody bead conjugate (Cell Signaling Technology) starting from 20 mg total protein, essentially according to the manufacturer’s protocol. Unbound peptides were removed by washing, and the captured peptides were eluted with a low pH buffer. Eluted peptides were analyzed by nanoflow LC–MS/MS.

### Nanoflow LC–MS/MS (SILAC)

Nanoflow LC–MS/MS was performed on an EASY-nLC system (Thermo) coupled to a Fusion Lumos Tribrid Orbitrap mass spectrometer (Thermo), operating in positive mode, and equipped with a nanospray source. Peptide mixtures were trapped on a ReproSil C18 reversed-phase column (Dr Maisch GmbH; column dimensions 1.5 cm × 100 μm, packed in-house) at a flow rate of 8 μl/min. Peptide separation was performed on ReproSil C18 reversed-phase column (Dr Maisch GmbH; column dimensions 15 cm × 50 μm, packed in-house) using a linear gradient from 0 to 80% B (A = 0.1% formic acid [FA]; B = 80% [v/v] AcN, 0.1% FA) in 70 or 120 min and at a constant flow rate of 250 nl/min. The column eluent was directly sprayed into the electrospray ionization source of the mass spectrometer. All mass spectra were acquired in profile mode. The resolution in mass spectrometry 1 (MS1) mode was set to 70,000 (automatic gain control [AGC]: 3E6), the *m/z* range 350 to 1700. Fragmentation of precursors was performed in data-dependent mode by high energy collisional dissociation (Top15) with a precursor window of 3.0 *m/z* and a normalized collision energy between 26.0 and 28.0; MS2 spectra were recorded in the ion trap. Singly charged precursors were excluded from fragmentation. Dynamic exclusion was set to 20 s, and the intensity threshold was set to 8.0E3. For the ubiquitinome analysis, a single LC–MS/MS run was performed for all immunoprecipitated peptide material from one sample.

### Data Analysis (SILAC)

Mass spectrometric raw data were analyzed using the MaxQuant software suite (version 1.6.15.0, https://www.maxquant.org/) (26) for identification and relative quantification of proteins. Precursor mass tolerances were set to 10 ppm, and fragment mass tolerance was set to 20 ppm for Orbitrap, 0.6 Da for ion-trap fragmentation data. A false discovery rate (FDR) of 0.01 for proteins and peptides and a minimum peptide length of six amino acids were required. The Andromeda search engine was used to search the MS/MS spectra against the *Mus musculus* UniProt database (63,756 entries; version: up_mouse_2017_09.fasta) concatenated with the reversed versions of all sequences and a contaminant database listing typical background proteins. A maximum of two missed cleavages were allowed. MS/MS spectra were analyzed using MaxQuant’s default settings for Orbitrap and ion trap spectra. The maximum precursor ion charge state used for searching was 7, and the enzyme specificity was set to trypsin. Further modifications were cysteine carbamidomethylation (fixed) as well as methionine oxidation and lysine ubiquitination (variable). The minimum number of peptides for positive protein identification was set to 2. Heavy-to-light (H:L) ratios were calculated using MaxQuant’s default settings, including a minimum ratio count of 2 for label-based protein quantification and of 1 for diGly peptide quantification. The minimum number of razor and unique peptides was set to 1. Only unique and razor nonmodified, methionine oxidized, and protein N-terminal acetylated peptides were used for protein quantitation. The minimal score for modified peptides was set to 40 (default value). The “requantify” option was selected in all cases. Only proteins that were identified and quantified in both duplicates and with consistent ratios were considered for further analysis. The proteingroups.txt, modificationspecificpeptides.txt, and GlyGly_(K)sites.txt output tables were all processed in R (version 4.1.0, The R Foundation). Reverse hits (decoys) and potential contaminants were filtered excluded from analysis. Normalized ratios of all replicate experiments were Log2 transformed, and these values were used in a one sample two-sided *t* test (mu = 0) to calculate *p* values. The modificationspecificpeptides.txt table was further adjusted by adding the diGly sites probability column from the GlyGly_(K)sites.txt table. Protein sets were further analyzed with Perseus (version 1.6.0.7, https://maxquant.net/perseus/) (27), through the use of ingenuity pathway analysis (IPA; QIAGEN, Inc, https://www.qiagenbioinformatics.com/products/ingenuity-pathway-analysis) (28), STRING analysis (https://string-db.org/) (29) and in-house developed software (30). For statistical testing of quantitative data, we used a one-sample *t* test in Perseus using 250 randomizations and further settings FDR = 0.05 and S0 = 0.5. Zipped MaxQuant data have been deposited to the PRIDE repository with the data identifier PXD035045.

### Sample Preparation and diGly Peptide Enrichment (Label-Free Quantitation)

Six 15 cm diameter plates of MEFs were grown and harvested for each condition (control shRNA, *Ube2d3* shRNA1, and *Ube2d3* shRNA2). Cells were washed twice with ice-cold PBS. Lysis, digestion, and enrichment of ubiquitinated peptides was performed according to the PTMScan HS K-ϵ-GG Remnant Magnetic Immunoaffinity Beads Kit protocol (Cell Signaling Technology; catalog no.: 34608). Briefly, cell pellets were lysed in 1× S-Trap lysis buffer, sonicated with a probe, and lysates were cleared by centrifugation. Equal protein amounts (2 mg per sample) were reduced with DTT, alkylated with chloroacetamide, and digested overnight at 37 °C with trypsin (Sigma–Aldrich, 1:10 dilution) using S-Trap Midi cartridges (ProtiFi). Peptides were eluted with elution buffers 1 to 3, after which 20 μg aliquots were taken for proteome analysis, the remainder being reserved for ubiquitinated peptide enrichment. All samples were dried in a Speedvac and stored at −80 °C until LC–MS/MS or further processing. Immunoaffinity purification of ubiquitinated peptides was performed with K-ϵ-GG Remnant Magnetic Immunoaffinity Beads using the manufacturer’s instructions. Ubiquitinated peptides were eluted twice with 50 μl 0.15% TFA and concentrated with C18 stage tips (Thermo Scientific; SP301).

### Nanoflow LC–MS/MS (Label-Free Quantitation)

Prior to MS analysis, peptides were reconstituted in 2% FA. Peptide mixtures were analyzed by nanoLC-MS/MS on an Q Exactive HF-X Hybrid Quadrupole-Orbitrap Mass Spectrometer equipped with an EASY-NLC 1200 system (Thermo Scientific). Samples were directly loaded onto the analytical column (ReproSil-Pur 120 C18-AQ, 1.9 μm, 75 μm × 500 mm, packed in-house). Solvent A was 0.1% FA/water, and solvent B was 0.1% FA/80% AcN. Samples were eluted from the analytical column at a constant flow of 250 nl/min. For single-shot proteome analysis, a 210-min gradient containing a linear 194-min increase from 6 to 26% solvent B was used, whereas a 144-min gradient containing a linear 124-min increase from 4 to 24% solvent B was used for ubiproteome analysis. MS settings were as follows: full MS scans (375–1500 *m/z*) were acquired at 60,000 resolution with an AGC target of 3 × 10^6^ charges and maximum injection time of 45 ms. Loop count was set to 20, and only precursors with charge state 2 to 7 were sampled for MS2 using 15,000 resolution (or 30,000 for ubiproteome), MS2 isolation window of 1.4 *m/z*, 1 × 10^5^ AGC target, a maximum injection time of 22 ms (or 54 ms for ubiproteome), and a normalized collision energy of 26.

### Data Analysis of Proteome and Ubiquitinated Peptides (Label-Free Quantitation)

Proteome and ubiproteome data (RAW files) were analyzed by label-free quantitation (LFQ) using MaxQuant (version 1.6.17.0) (31) with standard settings. Precursor mass tolerances were set to 20 ppm in the first search and 4.5 ppm in the main search, and fragment mass tolerances was set to 20 ppm. MS/MS data were searched against the mouse Swiss-Prot reviewed database (17,042 entries, release 2020_07). Carbamidomethylation on cysteine was specified as fixed modification, whereas methionine oxidation and protein N-terminal acetylation were set as variable modifications. Trypsin/p was specified as cleavage specificity, FDRs were set to 1% for both protein and peptide levels and in the case of diGly peptide enrichment experiments, and GG(K) was set as additional variable modification. LFQ intensities were Log2-transformed in Perseus (version 1.6.14.0) (27), after which proteins (in the case of proteome analysis) or ubiquitination sites (in the case of ubiproteome analysis) were filtered for at least three valid values (out of three total) in at least one condition. For ubiquitination sites, localization probability >0.75 was used as additional filter, and intensities were normalized by median subtraction. Missing values were replaced by an imputation-based normal distribution using a width of 0.3 and a downshift of 1.8. Differential proteins or ubiquitination sites were determined using a *t* test (cutoffs: *p* < 0.05 and LFQ differences log2 ≥ 1.0 and log2 ≤ −1.0). Annotated spectra have been deposited to MS-Viewer: For proteome, the link is: https://msviewer.ucsf.edu/prospector/cgi-bin/mssearch.cgi?report_title=MS-Viewer&search_key=3opswum57r&search_name=msviewer (key: 3opswum57r), and for the diGly dataset, the link is: https://msviewer.ucsf.edu/cgi-bin/mssearch.cgi?report_title=MS-Viewer&search_key=kapdokzmqp&search_name=msviewer (key: kapdokzmqp). Zipped MaxQuant data have been deposited to the PRIDE repository with the data identifier PXD035045.

### UBE2D3-TULIP2 Sample Preparation

UBE2D3-TULIP2 samples were prepared as previously described (21), UBE2D3 WT and C85A mutant were cloned in the TULIP2 and TULIP2ΔGG plasmids by Gateway cloning (Thermo Fisher Scientific). TULIP2 constructs were introduced in HeLa cells by lentiviral transduction, and positive clones were selected on 3 μg/ml puromycin.

Five 15 cm diameter plates of HeLa cells were grown up to 60 to 80% confluency. Expression of UBE2D3-TULIP2 constructs was induced with 1 μg/ml doxycycline for 24 h, and cells were untreated or treated for 5 h with 10 μM proteasome inhibitor MG132 (Sigma–Aldrich). Next, cells were washed twice with ice-cold PBS, scraped, and lysed in 10 ml guanidinium buffer (6 M guanidine–HCl, 0.1 M sodium phosphate, 10 mM Tris, pH 7.8). Samples were homogenized at room temperature by sonication using a tip sonicator (Q125 Sonicator; QSonica). Protein concentration was determined by using BCA Protein Assay Reagent (Thermo Scientific) and equalized accordingly.

Once equalized, lysates were supplemented with 5 mM β-mercaptoethanol and 50 mM imidazole (pH 7.8). For each sample 100 μl of nickel–nitrilotriacetic acid (Ni–NTA)–agarose beads (QIAGEN) were equilibrated with guanidinium buffer supplemented with 5 mM β-mercaptoethanol and 50 mM imidazole (pH 7.8), added to the cell lysates, and incubated overnight at 4 °C under rotation. Next, Ni–NTA beads were transferred with wash buffer 1 (6 M guanidine–HCl, 0.1 M sodium phosphate, 10 mM Tris, 10 mM imidazole, 5 mM β-mercaptoethanol, 0.2% Triton X-100, pH 7.8) to an Eppendorf LoBind tube (Eppendorf) and sequentially washed with wash buffer 2 (8 M urea, 0.1 M sodium phosphate, 10 mM Tris, 10 mM imidazole, 5 mM β-mercaptoethanol, pH 8), wash buffer 3 (8 M urea, 0.1 M sodium phosphate, 10 mM Tris, 10 mM imidazole, 5 mM β-mercaptoethanol, pH 6.3), and twice with wash buffer 4 (8 M urea, 0.1 M sodium phosphate, 10 mM Tris, 5 mM β-mercaptoethanol, pH 6.3). In every wash step, beads were allowed to equilibrate with the buffer for 15 min under rotation.

Ni–NTA beads were resuspended in one bead volume of elution buffer without imidazole (7 M urea, 0.1 M sodium phosphate, and 10 mM Tris [pH 7.0]) and incubated with 375 ng of rLys-C (Promega) for 5 h at 37 °C on a shaker at 1400 rpm. Subsequently, four volumes of 50 mM ammonium bicarbonate were added to the samples, and a second digestion using 500 ng trypsin (Promega) was done overnight at 37 °C and 1400 rpm for a complete digestion. Resulting peptides were purified and desalted using C-18 StageTips (32).

### MS Data Acquisition (UBE2D3-TULIP2)

UBE2D3 samples were analyzed in an Orbitrap Exploris 480 (Thermo Fisher Scientific) mass spectrometer coupled to an Ultimate 3000 UHPLC (Dionex). Digested peptides were separated using a 50 cm long fused silica emitter (FS360-75-15-N-5-C50; New Objective) in-house packed with 1.9 μm C18-AQ beads (Reprospher-DE, Pur, Dr Maisch) and heated to 50 °C in a column oven for electrospray ionization/Nano Spray (Sonation). Peptides were separated by liquid chromatography using a gradient from 2% to 32% AcN with 0.1% FA for 100 min followed by column reconditioning for 20 min. A lock mass of 445.12003 (polysiloxane) was used for internal calibration. Data were acquired in a data-dependent acquisition mode with a TopSpeed method with cycle time of 3 s with a scan range of 300 to 1600 *m/z* and resolutions of 60,000 and 30,000 for MS1 and MS2, respectively. For MS2, an isolation window of 1.6 *m/z* and a high energy collisional dissociation energy of 28% was applied. Precursors with a charge of 1 and higher than 6 were excluded from triggering MS2 as well as previously analyzed precursors with a dynamic exclusion window of 30 s.

### MS Data Analysis (UBE2D3-TULIP2)

MS data were analyzed using MaxQuant, version 1.6.14.0 according to Tyanova *et al.* (26) with the following modifications: maximum missed cleavages by trypsin was set to 4. The initial precursor maximum mass tolerances were set to 20 ppm in the first search and 4.5 ppm in the main search, and the fragment mass tolerances were set to 20 ppm. Searches were performed against an *in silico* digested database from the human proteome including isoforms and canonical proteins (96,850 entries; UniProt, June 8, 2020). Oxidation (M), acetyl (protein N-term), GlyGly (K), and Phospho (STY) were set as variable modifications with a maximum of 3. Carbamidomethyl (C) was disabled as fixed modification. LFQ was activated not enabling fast LFQ. FDRs at the peptide and protein level were 0.01. The match between runs feature was activated with default parameters.

MaxQuant output data were further processed in the Perseus Computational Platform v1.6.14 according to Tyanova *et al.* (27). LFQ intensity values were log2 transformed, and potential contaminants and proteins identified by site only or reverse peptide were removed. Samples were grouped in experimental categories, and proteins not identified in four of four replicates in at least one group were also removed. Missing values were imputed using normally distributed values with a 1.8 downshift (log2) and a randomized 0.3 width (log2) considering whole matrix values. Two-sided *t* tests were performed to compare groups. Analyzed data were exported from Perseus and further processed in Microsoft Excel 365 for comprehensive visualization. Volcano plots were generated using VolcanoseR (Joachim Goedhart and Martijn Luijsterburg) (33). Annotated spectra have been deposited to MS-Viewer: https://msviewer.ucsf.edu/cgi-bin/mssearch.cgi?report_title=MS-Viewer&search_key=lqf88o5m68&search_name=msviewer (key: lqf88o5m68).

### Quantitative Real-Time PCR

To determine gene expression levels, total RNA was isolated using TRIzol reagent (Ambion) and reverse transcribed into cDNA using the Tetro cDNA Synthesis kit (BIO-65043; Bioline) with Oligo(dT) primers. Quantitative real-time PCR was performed using the SensiFAST SYBR No-ROX kit (BIO-98020; Bioline) on a QuantStudio 5 Flex real-time PCR system (Thermo Fisher Scientific). *Hprt* was used as a reference for transcript expression. Transcripts were amplified using the following primers for mouse *Tspan8* forward: 5′-CTGACTGTGCAACTTATCA GG-3′ and mouse *Tspan8* reverse: 5′-GCCAGTCCAAAAGCAATTCC-3′; for mouse *Crabp1* forward: 5′-CGGAGATCAACTTCAAGGTCGG-3′ and mouse *Crabp1* reverse: 5′-CCCTCAAGAAGTGTCT GTGTGC-3′; for mouse *Hprt* forward: 5′-CTGGTGAAAGGACCTCTCG-3′ and mouse *Hprt* reverse: 5′-TGAAGTACTCATTATAGTCAAGGGCA-3′. Data were analyzed according to the 2-ΔCt methodology.

### Immunoprecipitation

For immunoprecipitation with ectopically expressed V5-tagged constructs, control (nontargeting shRNA) and UBE2D3-depleted HEK 293T cells were cotransfected with pcDNA3.1(+)-hemagglutinin (HA)-ubiquitin (34) and either pLX304-blast-V5 or pLX304-blast-RPS10-V5 (CCSB-Broad Lentiviral Expression Library hORFeome v8.1) (35). Or HEK 293T cells expressing pCDH-puro, pCDH-puro-UBE2D3 WT, or pCDH-puro-UBE2D3 C85A were transfected with pcDNA3.1(+)-HA-ubiquitin and either pLX304-blast-V5 or pLX304-blast-RPS10-V5. At 48 h post-transfection, cells were washed in ice-cold PBS and scraped in 450 μl lysis buffer (20 mM Tris–HCl [pH 7.5], 150 mM NaCl, and 0.5% Triton X), freshly supplemented with protease inhibitors (cOmplete; catalog no.: 4693124001, Roche), phosphatase inhibitors (PhosSTOP; catalog no.: 4906837001, Roche), 0.1 mM PMSF, 1 mM DTT, and 10 mM iodoacetamide. To each sample 150 μl B3 buffer (100 mM Tris–HCl [pH 8.0], 1 mM EDTA, 2% SDS) was added, and samples were sonicated. Then 900 μl lysis buffer was added, and samples were centrifuged for 20 min at maximum speed at 4 °C. Lysates were added to V5-conjugated Protein G Dynabeads (Invitrogen) and incubated overnight while rotating at 4 °C. Beads were washed four times with lysis buffer, and bound proteins were eluted with 2× sample buffer (100 mM Tris–HCl [pH 6.8], 4% SDS, and 20% glycerol) containing 10 mM DTT and boiling for 15 min at 100 °C. For endogenous immunoprecipitation experiments, control (nontargeting shRNA) and UBE2D3-depleted HEK 293T cells were transfected with pcDNA3.1(+)-HA-ubiquitin and the same procedure as aforementioned was followed, with the exception that the pulldown was done with RPS20 antibody–conjugated Protein G Dynabeads.

### Immunoblotting

Whole-cell lysates were prepared by scraping cells in SDS sample buffer (125 mM Tris [pH 6.8], 20% glycerol, 4% SDS). Lysates were sheared with a 25G needle and boiled for 10 min at 95 °C. Protein concentration was determined by standard BCA protein assay (Pierce). Equal amounts of protein were separated on precast polyacrylamide gels (Invitrogen). Immunoblotting was done according to standard methods using IRDye800CW- and IRDye680-labeled secondary antibodies for detection on the Odyssey Infrared imager (LI-COR) or using horseradish peroxidase–conjugated secondary antibodies for detection by enhanced chemiluminescence (Supersignal and Supersignal West Pico Plus; Thermo Scientific). Primary antibodies used were against UBE2D (A615, Boston Biochem, 1:2000 dilution or 4330S, CST, 1:500 dilution), V5 (R960-25, Invitrogen, 1:1000 dilution), HA (MMS-101R, Covance, 1:1000 dilution), FLAG (F3165, Sigma, 1:1000 dilution), RPS20 (ab133776, Abcam, 1:1000 dilution), Histone H3 (ab1791, Abcam, 1:10,000 dilution), CRABP1 (HPA017203, Sigma, 1:1000 dilution), TSPAN8 (A06997-1, SanBio, 1:1000 dilution), GAPDH (PA1-987, ThermoFisher, 1:5000 dilution), HSP90 α/β (sc-7947, Santa Cruz, 1:1000 dilution), β-actin (A5316, Sigma, 1:10,000 dilution), and γ-tubulin (T6557, Sigma, 1:10,000 dilution).

### Experimental Design and Statistical Rationale

The number of samples in the SILAC-based diGly proteomics was two, since one experimental condition (*Ube2d3* shRNA1) was studied with respect to its appropriate control (control shRNA). Six biological replicates of both sample and control were performed in a “forward” SILAC fashion, resulting in a total number of 12 samples. For every sample, the appropriate control was analyzed in the same experiment, that is, sample in the SILAC heavy channel and control in the SILAC light channel. Statistical analysis was performed using a two-sided one-sample *t* test, which is appropriate for SILAC H:L ratio analysis based on multiple replicates.

For all three experimental conditions in the LFQ diGly proteomics (control shRNA, *Ube2d3* shRNA1 and *Ube2d3* shRNA2), three biological replicates were used for comparison of protein or ubiquitinated peptide LFQ abundances, as this number of replicates is the minimum number of replicates required in order to be able to reliably use a two-sided Student’s *t* test for comparison of each shRNA cell line *versus* the WT control condition.

The total number of samples in the TULIP2 assays were 24, corresponding to the TULIP2 UBE2D-WT, TULIP2ΔGG UBE2D3-WT, and TULIP2 UBE2D3-C85A in four biological replicates, either treated or not treated with the proteasome inhibitor MG132. TULIP2ΔGG U BE2D3-WT and TULIP2 UBE2D3-C85A both serve as negative controls. Four replicates were chosen to have enough statistical power, while keeping the size of the project on a reasonable size. Statistical analysis was performed using two-sided *t* tests.

## Results

**Fig. 1:**
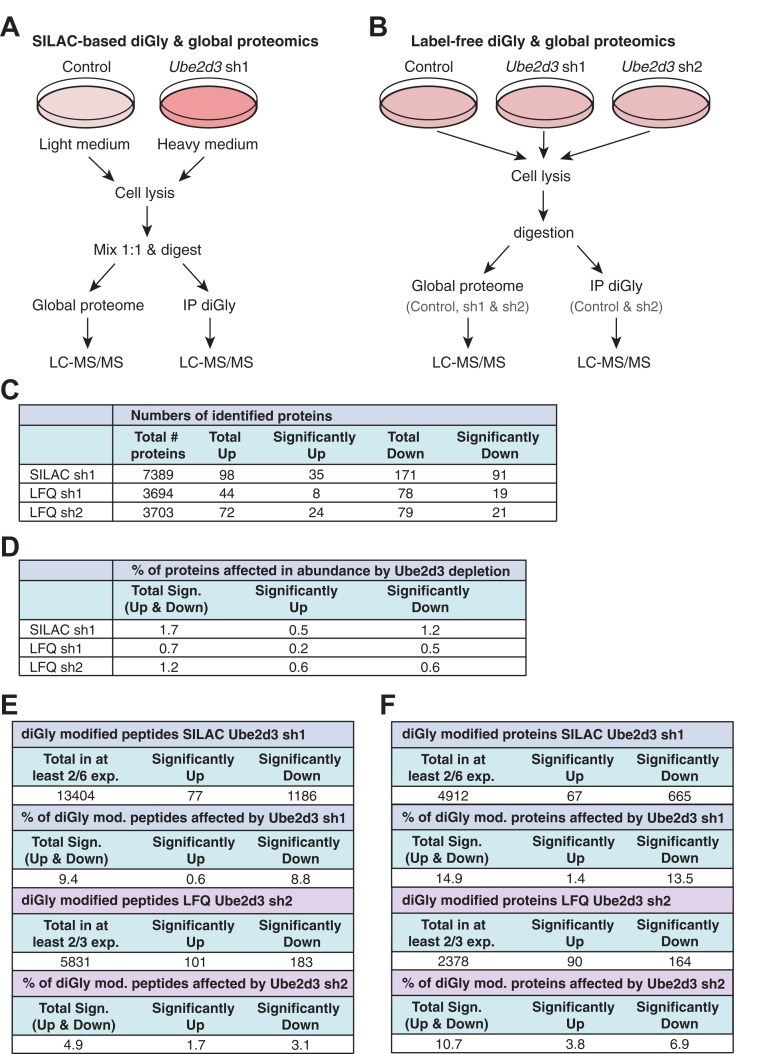
**Ubiquitinome and global proteome profiling to identify novel *in vivo* targets of UBE2D3.***A*, overview of the experimental approach used for SILAC-based quantitative ubiquitin diGly proteomics. Control and UBE2D3-depleted MEFs were cultured in SILAC medium with heavy or light isotopes. Cells were lysed, lysates were mixed in a 1:1 ratio (on total protein content), and digested. Both global proteome analysis and analysis for enriched ubiquitinated (diGly) peptides were performed for six biological replicates. *B*, setup LFQ global and diGly proteomics. Control and UBE2D3-depleted MEFs were lysed and digested. Global proteome analysis and analysis for enriched diGly peptides were performed for three biological replicates. *C*, table with numbers of proteins identified in the global proteomics experiments for SILAC *Ube2d3* sh1 (in at least two of six replicates) and LFQ *Ube2d3* sh1 and sh2 (in at least two of three replicates), including numbers of proteins that are upregulated or downregulated in abundance in UBE2D3-depleted cells. Protein abundance is significantly upregulated or downregulated when *p* ≤ 0.05. *D*, table with percentage of proteins affected in abundance by UBE2D3 depletion for SILAC *Ube2d3* sh1 and LFQ *Ube2d3* sh1 and sh2. The total percentage of proteins significantly upregulated, downregulated, and upregulated and downregulated in UBE2D3-depleted cells is shown. Significant when *p* ≤ 0.05. Upregulated for SILAC sh1: log2 ≥0.585 and downregulated for SILAC sh1: log2 ≤−0.585. Upregulated for LFQ sh1 and sh2: log2 ≥1.0; downregulated for LFQ sh1 and sh2: log2 ≤−1.0. *E*, table with diGly-modified peptides and percentages of diGly-modified peptides affected by UBE2D3 depletion. The *top part* shows the total number of identified diGly-modified peptides in at least two of six SILAC *Ube2d3* sh1 experiments and which of these are significantly upregulated or downregulated in their ubiquitination upon UBE2D3 depletion (significant: *p* ≤ 0.05; up: log2 ≥ 0.585; down: log2 ≤ −0.585), and the total percentage of diGly-modified peptides significantly upregulated, downregulated, or upregulated and downregulated in UBE2D3-depleted cells for SILAC *Ube2d3* sh1. The *bottom part* shows the total number of identified diGly-modified peptides in at least two of three LFQ *Ube2d3* sh2 experiments and which of these are significantly upregulated or downregulated in their ubiquitination upon UBE2D3 depletion (significant: *p* ≤ 0.05; up: log2 ≥ 1.0; down: log2 ≤ −1.0), and the total percentage of diGly-modified peptides significantly upregulated, downregulated, or upregulated and downregulated in UBE2D3-depleted cells for LFQ *Ube2d3* sh2. *F*, table with diGly-modified proteins and percentages of diGly-modified proteins affected by UBE2D3 depletion. The *top part* shows the total number of identified diGly-modified proteins in at least two of six SILAC *Ube2d3* sh1 experiments and which of these are significantly upregulated or downregulated in their ubiquitination upon UBE2D3 depletion (significant: *p* ≤ 0.05; up: log2 ≥ 0.585; down: log2 ≤ −0.585), and the total percentage of diGly-modified proteins significantly upregulated, downregulated, or upregulated and downregulated in UBE2D3-depleted cells for SILAC *Ube2d3* sh1. The *bottom part* shows the total number of identified diGly-modified proteins in at least two of three LFQ *Ube2d3* sh2 experiments and which of these are significantly upregulated or downregulated in their ubiquitination upon UBE2D3 depletion (significant: *p* ≤ 0.05; up: log2 ≥ 1.0; down: log2 ≤ −1.0), and the total percentage of diGly-modified proteins significantly upregulated, downregulated, or upregulated and downregulated in UBE2D3-depleted cells for LFQ *Ube2d3* sh2. LFQ, label-free quantitation; MEF, mouse embryonic fibroblast; SILAC, stable isotope labeling of amino acids in cell culture.

To identify *in vivo* UBE2D3 substrates, we investigated the effect of RNA interference–mediated UBE2D3 depletion on the global cellular proteome and ubiquitinome by combining affinity enrichment for ubiquitin diGly-modified peptides with SILAC-based proteomics. Control and *Ube2d3* shRNA1-transduced p53-deficient MEFs were differentially labeled by culturing them in SILAC media. Cells were cultured for two weeks in either the light medium with normal lysine and arginine (control cells) or the medium with heavy lysine and arginine isotopes (*Ube2d3* sh1 cells) to completely label all proteins with heavy amino acids. Subsequently, cells were harvested, lysed, and mixed at a 1:1 protein content ratio. The samples were digested and used for both global proteome analysis and ubiquitinome analysis after ubiquitin diGly-peptide enrichment (Fig. 1*A*). Experiments were performed as six biological replicates, all in a forward fashion and after confirming the reduction in UBE2D3 protein levels in heavy-labeled *Ube2d3* sh1 cells compared with light-labeled control cells (supplemental Fig. S1*A*). To exclude potential off-target effects of the shRNA used against *Ube2d3* (shRNA1) in the SILAC-based diGly proteomics analysis, we performed an independent validation including a second independent shRNA (shRNA2) targeting *Ube2d3*, in a label-free setting (LFQ). P53-deficient MEFs were transduced with control shRNA, *Ube2d3* shRNA1, or *Ube2d3* shRNA2 and used for global proteome analysis and for ubiquitinome analysis after diGly-peptide enrichment (Fig. 1*B* and supplemental Fig. S1*B*). Experiments were performed as three biological replicates.

**Fig. 2:**
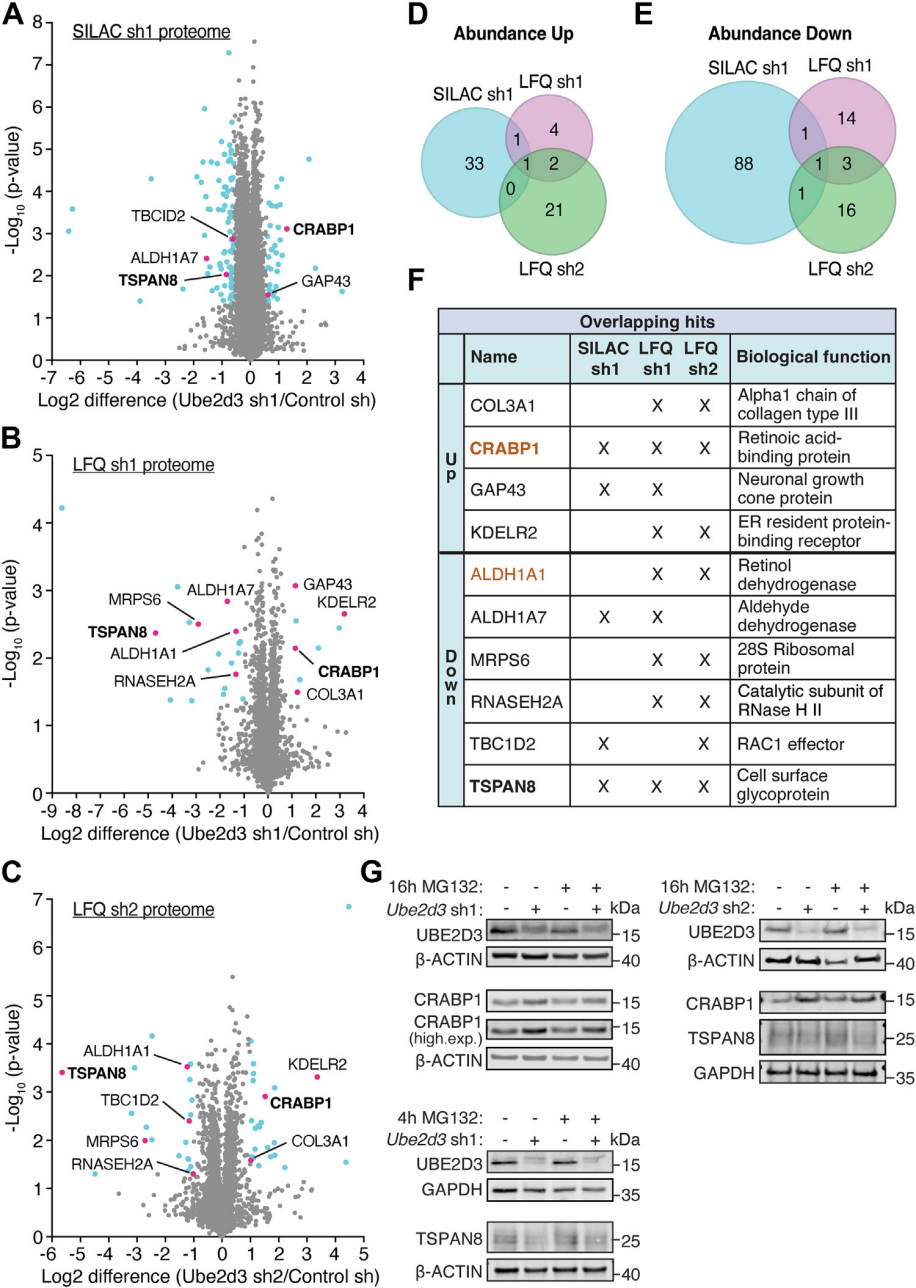
**UBE2D3 regulates the abundance of CRABP1 and TSPAN8.***A*, volcano plot showing changes in total protein levels upon UBE2D3 depletion with shRNA1 in the SILAC-based proteomics (n = 6). *Blue dots* represent proteins that are at least 1.5-fold upregulated or downregulated in abundance (log2 ≥ 0.585 and log2 ≤ −0.585, respectively) and have a *p* value of ≤0.05. *Red dots* indicate hits overlapping with LFQ proteomics with sh1 and/or sh2. *Bold proteins* overlap between SILAC *Ube2d3* sh1, LFQ *Ube2d3* sh1, and LFQ *Ube2d3* sh2. *B*, volcano plot showing changes in total protein levels upon UBE2D3 depletion with shRNA1 in LFQ (n = 3). *Blue dots* represent proteins that are at least twofold upregulated or downregulated in abundance (log2 ≥ 1.0 and log2 ≤ −1.0, respectively) and have a *p* value of ≤0.05. *Red dots* indicate hits overlapping with SILAC proteomics with *Ube2d3* sh1 and/or LFQ proteomics with *Ube2d3* sh2. *Bold proteins* overlap between SILAC *Ube2d3* sh1, LFQ *Ube2d3* sh1, and LFQ *Ube2d3* sh2. *C*, volcano plot showing changes in total protein levels upon UBE2D3 depletion with shRNA2 in LFQ (n = 3). *Blue dots* represent proteins that are at least twofold upregulated or downregulated in abundance (log2 ≥ 1.0 and log2 ≤ −1.0, respectively) and have a *p* value of ≤0.05. *Red dots* indicate hits overlapping with SILAC proteomics with *Ube2d3* sh1 and/or LFQ proteomics with *Ube2d3* sh1. *Bold proteins* overlap between SILAC *Ube2d3* sh1, LFQ *Ube2d3* sh1, and LFQ *Ube2d3* sh2. *D*, Venn diagrams illustrating the overlap between proteins that go up in abundance in SILAC *Ube2d3* sh1, LFQ *Ube2d3* sh1, and LFQ *Ube2d3* sh2. *E*, Venn diagrams illustrating the overlap between proteins that go down in abundance in SILAC *Ube2d3* sh1, LFQ *Ube2d3* sh1, and LFQ *Ube2d3* sh2. *F*, table of overlapping proteins in (*D* and *E*), including their biological functions. *Bold proteins* overlap between SILAC *Ube2d3* sh1, LFQ *Ube2d3* sh1, and LFQ *Ube2d3* sh2. *Orange colored proteins* are part of retinol metabolism and signaling pathways. *G*, immunoblot analysis of CRABP1, TSPAN8, and UBE2D3 protein levels in MEFs with or without depletion of UBE2D3 and untreated (DMSO) or treated with 10 μM of proteasome inhibitor MG132. For *Ube2d3* shRNA1 (sh1), cells were treated with 10 μM MG132 for 16 h for assessment of CRABP1 protein levels and for 4 h for assessment of TSPAN8 protein levels. For *Ube2d3* shRNA2 (sh2), cells were treated with 10 μM MG132 for 16 h for the assessment of CRABP1 and TSPAN8 protein levels. β-Actin and GAPDH serve as loading controls. Representative blots of three independent experiments are shown (two additional biological replicates for CRABP1 and TSPAN8 levels in *Ube2d3* sh1 cells and three additional biological replicates in *Ube2d3* sh2 cells can be found in supplemental Fig. S2*A*). DMSO, dimethyl sulfoxide; LFQ, label-free quantitation; MEF, mouse embryonic fibroblast; SILAC, stable isotope labeling of amino acids in cell culture.

The experiments were performed without addition of proteasome inhibitors, causing some targets of UBE2D3 to be potentially missed because of proteasomal degradation, but allowing assessment of the dynamics of protein abundance upon UBE2D3 depletion. For the SILAC-based proteomics, typically over 6300 proteins were identified per experiment (supplemental Fig. S1*C* and supplemental Table S1). Overall, on average over the six replicates, 130 proteins were ≥1.5-fold increased in abundance, whereas 195 proteins were ≥1.5-fold decreased in abundance upon UBE2D3 depletion, per experiment (supplemental Fig. S1*C*). For 35 of these proteins, the increase was statistically significant (*p* ≤ 0.05), and for 91 proteins, their decrease was statistically significant (Figs. 1*C* and 2*A*; supplemental Table S2). This translates into UBE2D3 significantly affecting the levels of ±1.7% of the total number of detected proteins, of which ±0.5% increased in abundance and ±1.2% decreased in abundance (Fig. 1*D*). For the LFQ proteomics, the results show that with both shRNAs around 3700 proteins were identified in at least two of three experiments (Fig. 1*C* and supplemental Tables S4 and S5). A total of eight proteins were significantly increased in abundance, and 19 proteins were significantly decreased in abundance in *Ube2d3* sh1-transduced cells. In *Ube2d3* sh2-transduced cells, 24 proteins were significantly increased in abundance and 21 proteins were significantly decreased in abundance (Figs. 1*C* and 2, *B* and *C*; supplemental Table S6). Consistent with the SILAC-based proteome data obtained with *Ube2d3* sh1, also these LFQ-proteome data show that UBE2D3 only regulates the abundance of a small percentage of the total number of identified proteins (Fig. 1*D*). Combined, the results of the SILAC-based and label-free approaches indicate that UBE2D3 not only decreases the levels of certain proteins by for instance promoting their degradation but also (indirectly) increases the levels of certain other proteins. However, the low numbers of proteins affected indicate that UBE2D3 only has a modest role in regulating protein abundance.

SILAC-based ubiquitinome analysis revealed over 19,000 diGly peptides over all experiments. In total, almost 500 peptides were ≥1.5-fold increased in ubiquitination, whereas over 3500 peptides were ≥1.5-fold decreased in ubiquitination upon UBE2D3 depletion (supplemental Fig. S1*D*). As can be expected with depleting an E2 enzyme that would most likely result in loss of ubiquitination of its targets, the number of peptides that decreased in ubiquitination is substantially higher than the number of peptides that increased in ubiquitination. Over 13,000 peptides were diGly modified in at least two of six experiments, of which 77 peptides were significantly (*p* ≤ 0.05) increased in their ubiquitination and 1186 peptides were significantly decreased in their ubiquitination in UBE2D3-depleted cells (Fig. 1*E* and supplemental Tables S7–S9). This corresponds to ±4900 diGly-modified proteins, of which 67 were significantly increased (≥1.5 fold; *p* ≤ 0.05) in their ubiquitination and 665 were significantly decreased in their ubiquitination in UBE2D3-depleted cells (Fig. 1*F*). Thus, we found UBE2D3 to significantly affect the ubiquitination status of ±9.5% of all detected diGly-modified peptides, corresponding to ±15% of the total amount of diGly-modified proteins. Of these, 0.6% of the diGly-modified peptides and 1.4% of the diGly-modified proteins were increased in their ubiquitination, and 8.8% of the diGly-modified peptides and 13.5% of the diGly-modified proteins were decreased in their ubiquitination upon depletion of UBE2D3 (Fig. 1, *E* and *F*). Label-free ubiquitinome analysis revealed 5831 diGly-modified peptides over all experiments (Fig. 1*E*). A total of 101 peptides, representing 90 unique proteins, were significantly increased in their ubiquitination upon UBE2D3 depletion, whereas 183 peptides, representing 164 unique proteins, were decreased in their ubiquitination (≥2.0-fold; *p* ≤ 0.05) (Fig. 1, *E* and *F* and supplemental Tables S10 and S11). These results show that UBE2D3 significantly affects the ubiquitination of ±5% of all detected diGly-modified peptides, corresponding to ±11% of all diGly-modified proteins, in at least two of three experiments (Fig. 1, *E* and *F*). Thus, in contrast to the promiscuous behavior of UBE2D3 seen under *in vitro* conditions, *in vivo* UBE2D3 is a quite selective E2 enzyme, only affecting a small portion of the total number of identified diGly-modified proteins and peptides.

### Global Proteome Changes Upon UBE2D3 Depletion

Ubiquitination of proteins by UBE2D3 might change the stability of its direct or indirect target proteins by affecting their proteasomal degradation. Direct targets of UBE2D3 are proteins that are directly ubiquitinated through association of UBE2D3 with an E3 ligase and subsequent transfer of the ubiquitin to the target protein, whereas indirect targets of UBE2D3 are not directly subject of UBE2D3-mediated ubiquitination, but are indirectly affected in their ubiquitination and/or stability as a result of UBE2D3-mediated ubiquitination of other proteins. Therefore, we first focused on the 126 proteins that significantly changed ≥1.5-fold in abundance upon UBE2D3 depletion in the SILAC-based global proteomics (Fig. 2*A* and supplemental Table S2). As the quantitative accuracy of SILAC MS is higher than that of label-free MS, we increased the cutoff for the LFQ-proteome data and selected proteins with a ≥2-fold and significant (*p* ≤ 0.05) change in abundance for further analysis (Fig. 2, *B* and *C* and supplemental Table S6).

When we overlaid the hits from the SILAC proteomics (SILAC sh1), the LFQ with *Ube2d3* sh1 (LFQ sh1), and the LFQ with *Ube2d3* sh2 (LFQ sh2), we found only CRABP1 to be significantly increased in abundance in all three experiments, and only TSPAN8 to be significantly decreased in abundance in all three experiments (Fig. 2, *D*–*F*). When assessing the overlap between 2 out of 3 SILAC or LFQ experiments, we found three additional proteins that increased in abundance and five additional proteins that decreased in abundance (Fig. 2, *D*–*F* and supplemental Table S3). In general, hits from the SILAC-based proteomics with *Ube2d3* sh1 that were not overlapping with the LFQ sh1 and LFQ sh2 experiments were either not identified in the LFQ experiments or were altered just below the cutoffs that we set.

CRABP1 is a protein that responds to retinol (vitamin A) signaling by binding to RA (a metabolite of retinol). In the nucleus, RA binds and activates RA receptors, eventually resulting in transcription of target genes and thereby regulating differentiation and proliferation (36). CRABP1 sequesters RA in the cytoplasm and thereby prevents activation of gene expression (36, 37, 38). As CRABP1 levels increase upon UBE2D3 depletion in the SILAC and LFQ diGly proteomics data (Fig. 2, *A*, *B*, *C*, and *F*), UBE2D3 might promote degradation of CRABP1 and thereby impact RA signaling. To address this hypothesis, we assessed CRABP1 protein levels in immunoblots of protein lysates from control cells and UBE2D3-depleted cells, treated with and without the proteasome inhibitor MG132 (Fig. 2*G* and supplemental Fig. S2*A*). Indeed, we found CRABP1 protein levels to be increased upon UBE2D3 depletion, thereby validating the proteomics results. However, MG132 treatment of up to 16 h, which was the maximum duration tolerated by the cells, did not lead to clearly visible CRABP1 protein stabilization, which precluded us from confirming that UBE2D3 promotes proteasomal degradation of CRABP1 (Fig. 2*G* and supplemental Fig. S2, *A* and *B*). Nevertheless, qRT–PCR analysis indicated that the increased CRABP1 protein levels upon UBE2D3 depletion are not accompanied by increased *Crabp1* mRNA levels, indicating that UBE2D3 regulates CRABP1 on protein level (supplemental Fig. S2*C*).

TSPAN8 is a cell surface glycoprotein belonging to the transmembrane protein family of tetraspanins that function in intracellular signal transduction, cell adhesion, and motility. It forms a complex with integrins, which promote cell–cell and cell–matrix adhesion (39, 40, 41, 42, 43). A decrease in TSPAN8 level in UBE2D3-depleted cells suggests that UBE2D3 indirectly regulates TSPAN8 levels (Fig. 2, *A*, *B*, *C*, and *F*). To independently validate the impact of UBE2D3 on TSPAN8 protein levels and to investigate if this is dependent on the proteasome, we assessed TSPAN8 protein levels in immunoblots of protein lysates from control cells and UBE2D3-depleted cells, treated with and without the proteasome inhibitor MG132 (Fig. 2*G* and supplemental Fig. S2*A*). Indeed, UBE2D3-depleted cells express lower levels of TSPAN8 protein, confirming the proteomics results. However, MG132 barely affected TSPAN8 protein levels over the duration of treatment, prohibiting us from determining the involvement of proteasomal degradation in the regulation of TSPAN8 levels by UBE2D3 (Fig. 2*G* and supplemental Fig. S2*A*). However, qRT–PCR analysis indicated that the decreased TSPAN8 protein levels upon UBE2D3 depletion are not because of decreased *Tspan8* mRNA levels. Upon the expression of *Ube2d3* shRNA1 TSPAN8, transcript levels were even significantly increased over three independent biological replicates. Thus, the reduced TSPAN8 protein levels in UBE2D3-depleted cells seem to result from regulation at the protein level (supplemental Fig. S2*D*).

### Ubiquitinome Analysis Identifies Increased Protein Ubiquitination in UBE2D3-Depleted Cells

**Fig. 3:**
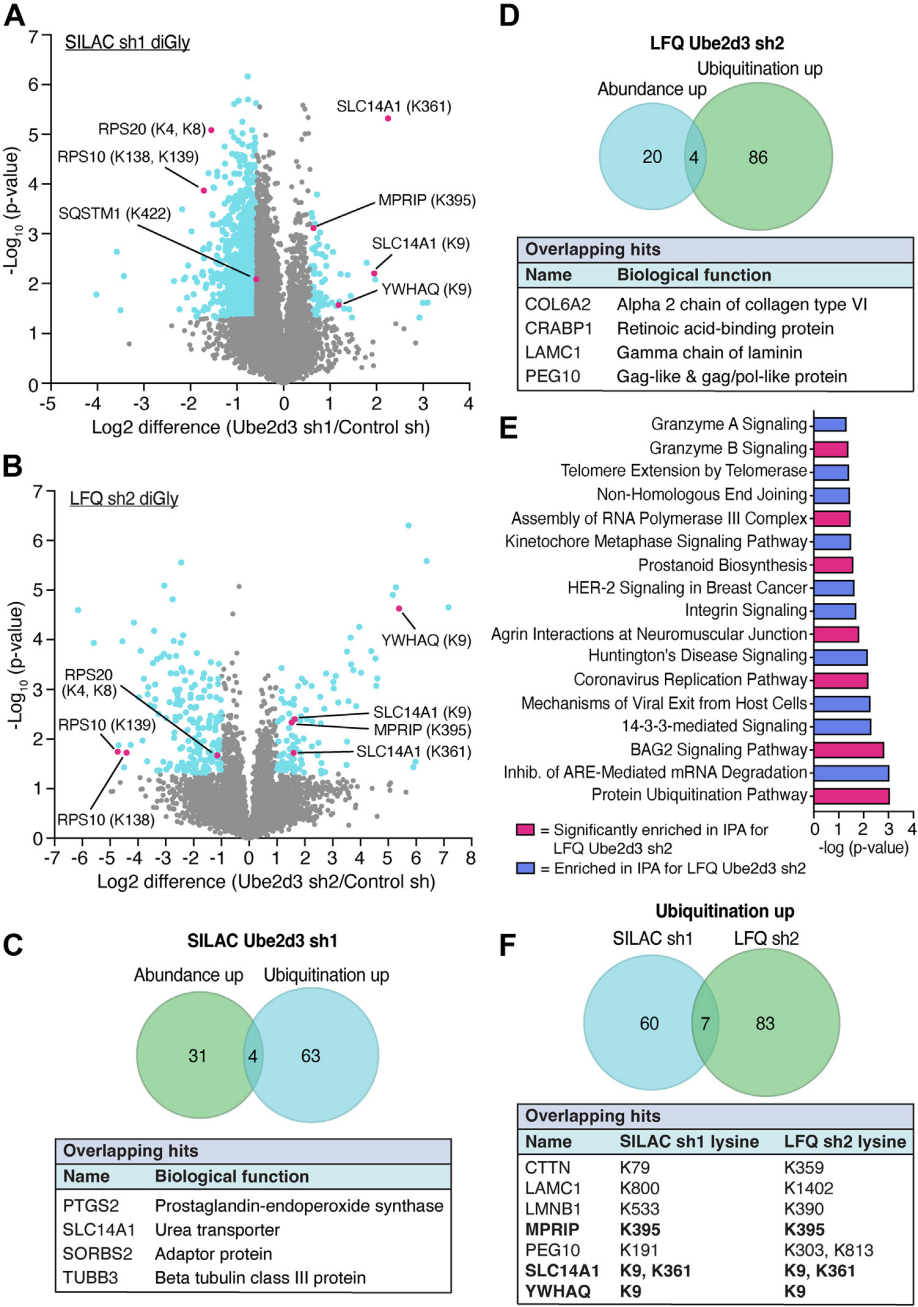
**DiGly sites upregulated upon UBE2D3 depletion.***A*, volcano plot showing the log2 fold changes of diGly (ubiquitinated) peptides upon UBE2D3 depletion with sh1 in SILAC-based diGly proteomics (n = 6). *Blue dots* represent peptides that are at least 1.5× upregulated or downregulated in their ubiquitination (log2 ≥ 0.585 and log2 ≤ −0.585) and have a *p* value of ≤0.05. *Red dots* represent top hits, including the modified lysine(s), which are overlapping with LFQ *Ube2d3* sh2 hits. *B*, volcano plot showing the log2 fold changes of diGly peptides upon UBE2D3 depletion with sh2 in LFQ proteomics (n = 3). *Blue dots* represent peptides that are at least 2× uprregulated or downregulated in their ubiquitination (log2 ≥ 1.0 and log2 ≤ −1.0) and have a *p* value of ≤0.05. *Red dots* represent top hits, including the modified lysine, that are overlapping with SILAC *Ube2d3* s1 hits. *C*, Venn diagram illustrating the overlap between proteins that go up in abundance and ubiquitination in SILAC *Ube2d3* sh1. The table below shows the overlapping hits and their biological function. *D*, Venn diagram illustrating the overlap between proteins that go up in abundance and ubiquitination in LFQ with *Ube2d3* sh2. The table below shows the overlapping hits and their biological function. *E*, ingenuity pathway analysis of diGly peptides upregulated upon UBE2D3 depletion. Bar plots show the top significantly enriched pathways from SILAC *Ube2d3* sh1 that overlap with LFQ *Ube2d3* sh2. *F*, Venn diagram illustrating the overlap between proteins that go up in ubiquitination in SILAC *Ube2d3* sh1 and LFQ *Ube2d3* sh2. The table below shows the overlapping hits, including on which lysine they are ubiquitinated. *Bold hits* are ubiquitinated on the same lysine in the SILAC *Ube2d3* sh1 experiments as in the LFQ *Ube2d3* sh2 experiments. LFQ, label-free quantitation; SILAC, stable isotope labeling of amino acids in cell culture.

To identify novel direct and indirect targets of UBE2D3 *in vivo*, we subsequently focused on the ubiquitinome changes occurring upon UBE2D3 depletion in our SILAC-based diGly proteomics and LFQ diGly proteomics data, represented by diGly peptides with a normalized ratio of ≥1.5-fold (SILAC) or ≥2-fold (LFQ) that were significantly changed (*p* ≤ 0.05) (Fig. 3, *A* and *B* and supplemental Tables S7–S11).

First, we examined the 67 (SILAC *Ube2d3* sh1) and 90 (LFQ *Ube2d3* sh2) proteins that were significantly increased ≥1.5-fold (SILAC) or ≥2-fold (LFQ) in their ubiquitination upon UBE2D3 depletion and most likely represent indirect targets of UBE2D3 (Fig. 1*F* and supplemental Tables S9 and S11). Of these, four proteins (PTGS2, SLC14A1, SORBS2, and tubulin beta-3 chain (TUBB3) increased in abundance in SILAC *Ube2d3* sh1 and four proteins (COL6A2, CRABP1, laminin subunit gamma 1 (LAMC1), and PEG10) increased in abundance in LFQ *Ube2d3* sh2 (Fig. 3, *C* and *D* and supplemental Table S3). Hence, the observed increase in their ubiquitination could be a consequence of their increased abundance. However, it could also reflect a functional role in which for instance the ubiquitination of these proteins interferes with their degradation, causing an increase in their abundance. CRABP1 was also found increased in ubiquitination and abundance in the SILAC diGly proteomics with shRNA1, but the change in ubiquitination was not statistically significant (supplemental Tables S2 and S7). Vice versa, TUBB3 was increased both in ubiquitination and abundance in the SILAC sh1 proteomics, whereas in the LFQ sh2 proteomics, TUBB3 was also increased in abundance, but its ubiquitination status remains unassessed as no diGly peptides were detected for TUBB3 (supplemental Tables S2, S6, S9, and S10).

Furthermore, while we do not necessarily expect proteins that become more ubiquitinated upon UBE2D3 depletion to be direct targets of UBE2D3, we did find 5 of 67 proteins identified in the SILAC-based diGly proteomics to be known UBE2D3 interactors (from *BioGRID*, *IntAct*, *MINT*, *STRING*, and *UniProt* databases) (supplemental Fig. S3*A* and supplemental Table S3). These are the E2 enzyme UBE2S, the E3 ligases ARIH1, DZIP3, and UHRF1, and the F-box protein FBXW11 (or β-TrCP2), a substrate-binding subunit of an SCF (Skp1–cullin–F-box) E3 ligase. UBE2S and UBE2D3 were shown to cooperate in the ubiquitination of APC/C substrates, such as cyclin B, *in vitro* (44). Furthermore, UBE2D3 was shown to function together with the E3 ligases ARIH1 and SCF (SCF^β-TrCP^ and SCF^FBW7^) in ubiquitination of targets, such as DCNL1, β-catenin, Myc, and IκBα (44, 45, 46, 47, 48, 49). The abundance of these five proteins (UBE2S, ARIH1, DZIP3, UHRF1, and FBXW11) was not changed upon UBE2D3 depletion (supplemental Table S1), suggesting that their increased ubiquitination in UBE2D3-depleted conditions has no effect on their proteasomal degradation but might have a regulatory consequence or be without effect. We also found three proteins with increased ubiquitination upon UBE2D3 knockdown in the LFQ diGly proteomics to be among known UBE2D3 interactors: the two E3 ligases HERC2 and UBE4B and the ubiquitin domain–containing protein UBTD1 (supplemental Fig. S3*B* and supplemental Table S3). HERC2 and UBE2D3 ubiquitinate BRCA1 *in vitro* (50), whereas UBE4B and UBE2D3 were shown to ubiquitinate the HTLV-1 Tax oncoprotein and FEZ1, which functions in axonal outgrowth and fasciculation (51, 52). In addition, UBTD1 interacts with UBE2D3 and β-TrCP in the β-TrCP–UBE2D3–β-catenin complex and promotes β-TrCP–YAP interaction and YAP ubiquitination (53). How UBE2D3 inhibits ubiquitination of these E3 ligases, UBE2S and UBTD1, and with what consequences, is unclear at this point and would be interesting to further address.

There is no overlap between known UBE2D3 interactors with increased ubiquitination in the SILAC *Ube2d3* sh1 diGly experiments and those in the LFQ *Ube2d3* sh2 diGly experiments, because for the UBE2D3 interactors with increased ubiquitination in the SILAC sh1 experiments, no diGly-modified peptides were detected in the LFQ sh2 experiments and vice versa. Therefore, we could not evaluate potential changes in ubiquitination. Except for UHRF1, which was increased in ubiquitination at K196 in both the SILAC *Ube2d3* sh1 and LFQ *Ube2d3* sh2 experiments, but which did not meet the cutoff for statistical significance in LFQ sh2, and was therefore not included in further analysis.

IPA of the 67 and 90 proteins that were more ubiquitinated upon depletion of UBE2D3, in the SILAC-based and LFQ diGly proteomics, respectively, revealed the ubiquitin signaling, cell adhesion and signaling, and apoptosis pathways as the top enriched overlapping pathways between SILAC sh1 and LFQ sh2 hits (Fig. 3*E* and supplemental Tables S12–S14). The cytoskeletal proteins alpha-actinin 4 (ACTN4), tubulin beta-3 chain (TUBB3), and tubulin beta-4B chain (TUBB4B), the regulators of the actin cytoskeleton Rho-associated protein kinase 1 (ROCK1) and myosin phosphatase Rho-interacting protein (MPRIP), and Src substrate cortactin (CTTN) are the main factors from our data that act in cell adhesion pathways. Cell adhesion is critical in cell migration and tissue development (54, 55). Disruption could lead to pathologies and plays an important role in cancer metastasis (56). By regulating these factors, next to TSPAN8, which is also involved in cell adhesion, UBE2D3 could potentially play a role in cell adhesion–regulated pathologies.

Of the 90 proteins that were significantly increased in their ubiquitination in the LFQ sh2 experiments, seven proteins were also identified as significantly increased in ubiquitination in the SILAC sh1 experiments (Fig. 3*F*). Of these seven proteins, three proteins were found to be modified on the same lysine in the SILAC and LFQ diGly experiments, further supporting that these proteins are potential new indirect targets of UBE2D3. Among these, SLC14A1 was also increased in abundance upon UBE2D3 depletion in the SILAC sh1 condition (Fig. 3*C*; it was not picked up in the LFQ sh2 global proteomics); therefore, the observed increase in ubiquitination could potentially be a consequence of increased abundance. However, for MPRIP and 14-3-3θ (YWHAQ), no change in abundance was observed, further supporting that these proteins are new indirect ubiquitination targets of UBE2D3. MPRIP is a filamentous actin-binding protein that can affect the integrity of the actin cytoskeleton (57). MPRIP ubiquitination at K395 (K396 human) is increased upon UBE2D3 depletion in both the SILAC sh1 and the LFQ sh2 condition. This lysine is located in the second Pleckstrin homology domain of MPRIP. Pleckstrin homology domains are known to promote recruitment of proteins to membranes. Potentially, by regulating ubiquitination of MPRIP at K395, UBE2D3 could affect interaction of MPRIP with other proteins and their recruitment to certain cellular compartments. 14-3-3 proteins are phosphoserine- and phosphothreonine-binding proteins that regulate almost every process in the cell. 14-3-3 proteins generally function as adaptors or chaperones and interact with many different proteins, such as kinases, phosphatases, and receptors (58, 59). UBE2D3 depletion results in increased ubiquitination of 14-3-3θ at K9, which is located in the first alpha-helix of the protein. 14-3-3 proteins can form dimers through the first two helices of one monomer and the fourth helix of the second monomer (58). Possibly, by affecting ubiquitination of 14-3-3 θ at K9, UBE2D3 could interfere with homodimerization or heterodimerization of 14-3-3θ. However, more research will be needed to determine how these indirect targets of UBE2D3 are ubiquitinated and what the potential consequences are.

### Ubiquitinome Analysis Identifies Decreased Protein Ubiquitination in UBE2D3-Depleted Cells

**Fig. 4:**
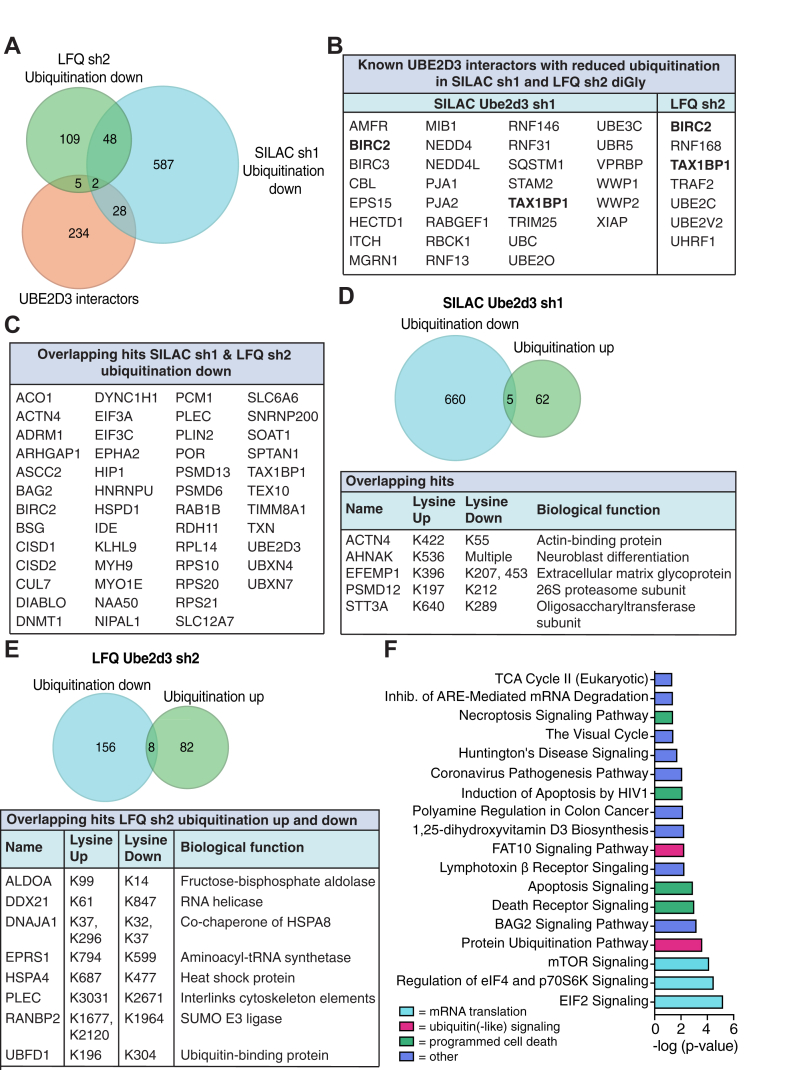
**DiGly sites downregulated upon UBE2D3 depletion.***A*, Venn diagrams illustrating the overlap between known UBE2D3 interactors (*BioGRID*, *IntAct*, *MINT*, *STRING*, and *UniProt* databases) and proteins significantly downregulated in their ubiquitination in the SILAC *Ube2d3* sh1 and LFQ *Ube2d3* sh2 experiments. *B*, table showing the overlap between known UBE2D3 interactors and proteins significantly reduced in their ubiquitination upon UBE2D3 depletion in the SILAC sh1 and LFQ sh2 datasets. In *bold* are the overlapping proteins found in both the SILAC *Ube2d3* sh1 and LFQ *Ube2d3* sh2 data. *C*, table showing the overlapping hits between proteins that go down in ubiquitination in SILAC with *Ube2d3* sh1 and LFQ with *Ube2d3* sh2. *D*, Venn diagram illustrating the overlap between proteins that go up and down in ubiquitination in SILAC *Ube2d3* sh1. The table below shows the overlapping hits, the lysines on which they are modified, and the biological function of these proteins. *E*, Venn diagram illustrating the overlap between proteins that go up and down in ubiquitination in LFQ *Ube2d3* sh2. The table below shows the overlapping hits, the lysines on which they are modified, and the biological function of these proteins. *F*, ingenuity pathway analysis of the 50× overlapping proteins between SILAC *Ube2d3* sh1 and LFQ *Ube2d3* sh2 that were significantly downregulated in their ubiquitination upon UBE2D3 depletion. Bar plots show the top 18 significantly enriched pathways. LFQ, label-free quantitation; SILAC, stable isotope labeling of amino acids in cell culture.

To identify novel direct targets of UBE2D3, we focused on the peptides that were less ubiquitinated in UBE2D3-depleted cells. From the 665 proteins that were on average reduced in their ubiquitination over all SILAC-based diGly proteomics experiments, 50 proteins were also found less ubiquitinated in the LFQ sh2 proteomics and 30 proteins overlap with previously identified UBE2D3 interactors (Fig. 4, *A*–*C* and supplemental Tables S3, S9, and S11). From the 164 proteins that were on average reduced in their ubiquitination over all three LFQ *Ube2d3* sh2 diGly proteomics experiments, seven proteins are known UBE2D3 interactors (Fig. 4, *A* and *B* and supplemental Tables S3 and S11). Most of these proteins are E3 ligases. One of these being CBL, for which UBE2D3 was previously shown to both promote autoubiquitination and substrate ubiquitination *in vitro* (60). Our data suggest that this autoubiquitination occurs on lysine 519, as ubiquitination on this lysine is significantly decreased upon UBE2D3 depletion (supplemental Table S9). Furthermore, CBL was reported to ubiquitinate the epidermal growth factor receptor (EGFR) together with UBE2D2/UBE2D3 (60, 61, 62), and depletion of CBL or UBE2D3 resulted in decreased EGFR ubiquitination (60). Indeed, the SILAC sh1 data also show significantly decreased ubiquitination of EGFR upon UBE2D3 depletion (supplemental Table S9), thus supporting the previous findings. By promoting (auto)ubiquitination of E3 ligases such as CBL, UBE2D3 could potentially be involved in regulating their activity.

We also identified sequestome-1 (SQSTM1/p62), which is known to interact with UBE2D3 and to be ubiquitinated at K420 by UBE2D3 to activate its function as an autophagy receptor (63). Human and mouse SQSTM1 share ±90% amino acid identity, with lysine 420 in human SQSTM1 corresponding to lysine 422 in mouse SQSTM1. In our SILAC-based diGly proteomics dataset SQSTM1(K422), ubiquitination is ±2-fold decreased in UBE2D3-depleted cells (Fig. 3*A* and supplemental Table S9), indicating that our data are in line with, and support, previous findings on SQSTM1 ubiquitination by UBE2D3. Unfortunately, in the LFQ diGly proteomics, no diGly peptide with lysine 422 was detected. In addition, we also identified the chaperone HSPA5 as one of the significant hits that is decreased in its ubiquitination (supplemental Table S9). SQSTM1 and HSPA5 are both part of the autophagic PQC pathway, a pathway that removes misfolded proteins and their aggregates *via* lysosomal degradation. HSPA5 binds to these protein aggregates and is recruited by SQSTM1. SQSTM1 delivers HSPA5 and its cargo to autophagosomes, which subsequently fuse with lysosomes to induce lysosomal degradation (64). By regulating the ubiquitination of these factors and the activity of SQSTM1, UBE2D3 might affect degradation of misfolded proteins *via* autophagic PQC.

Of the known UBE2D3 interactors, two proteins, TAX1BP1 and BIRC2, are reduced in their ubiquitination in both the SILAC *Ube2d3* sh1 and LFQ *Ube2d3* sh2 diGly proteomics (Fig. 4*B*). TAX1BP1 and BIRC2 are both involved in regulation of NF-κB signaling (65, 66, 67, 68). Interestingly, TAX1BP1 is an autophagy receptor that has also been proposed to function downstream of SQSTM1, promoting autophagosome formation and degradation of protein aggregates (69, 70). This adds another potential layer of regulation by UBE2D3, in which it might also ubiquitinate TAX1BP1, next to SQSTM1 and HSPA5, and thereby play a role in degradation of ubiquitinated proteins/protein aggregates by autophagy. Thus, our approaches not only successfully identified and confirmed previously known targets of UBE2D3 but are also a useful resource for establishing to what extent *in vitro* demonstrated ubiquitination activities of UBE2D3 also represent ubiquitination events with dependency on UBE2D3 *in vivo*. Moreover, they identified >700 novel direct or indirect *in vivo* targets of ubiquitination by UBE2D3.

Intriguingly, we also found five and eight proteins, in respectively, the SILAC *Ube2d3* sh1 and LFQ *Ube2d3* sh2 diGly proteomics, that both increased and decreased in their ubiquitination (on different lysine residues) following UBE2D3 depletion. These proteins have different biological functions, for example, in protein degradation, post-translational protein modification, and cytoskeleton support (Fig. 4, *D* and *E* and supplemental Table S3). Potentially, ubiquitination of these proteins on one lysine residue by UBE2D3 could inhibit or block ubiquitination by other enzymes on another lysine residue. However, further investigation is needed to understand the mechanisms and biological relevance associated with this differential ubiquitination.

### UBE2D3 Depletion Impairs Ubiquitination of Factors in mRNA Translation Pathways

As we identified a considerable number of proteins that were significantly reduced in their ubiquitination upon UBE2D3 depletion, we used IPA to understand in which biological processes these proteins work and whether specific processes were impacted. IPA analysis on the 50 proteins that were significantly less ubiquitinated in both SILAC *Ube2d3* sh1 and LFQ *Ube2d3* sh2 diGly proteomics revealed 18 significantly enriched pathways (Fig. 4, *A*, *C*, and *F* and supplemental Table S15). These mostly belong to mRNA translation and degradation, protein degradation/ubiquitin(-like) signaling, and programmed cell death processes. Furthermore, STRING analysis on the 50 proteins reduced in ubiquitination and overlapping between SILAC sh1 and LFQ sh2, also identified a network of proteins involved in mRNA translation and degradation (supplemental Fig. S4*A*). We also evaluated if the decreased ubiquitination of these 50 proteins upon UBE2D3 depletion could be a consequence of decreased abundance. However, we found no indication for this as there was no overlap between these 50 proteins and the proteins decreasing in abundance in the corresponding global proteome data from SILAC sh1 and LFQ sh2 (supplemental Fig. S4, *B* and *C*). The top hits that were affected by UBE2D3 depletion with both sh1 and sh2, and are related to mRNA translation, are the 40S ribosomal proteins RPS10 and RPS20. Both were >2.5-fold reduced in their ubiquitination upon UBE2D3 knockdown (Fig. 3, *A* and *B* and supplemental Tables S9 and S11). RPS10 and RPS20 ubiquitination plays an important role in the RQC pathway (22, 23, 24). Also, other proteins acting in the RQC pathway, such as the key components listerin (LTN1) and VCP/p97, were significantly decreased in their ubiquitination in the SILAC sh1 data (supplemental Table S9). Despite these changes not reaching statistical significance in the LFQ sh2 data (supplemental Table S10), this may point toward a broader impact of UBE2D3 on the RQC pathway.

### TULIP2 Confirms RPS10 and RPS20 as Direct Substrates of UBE2D3

**Fig. 5:**
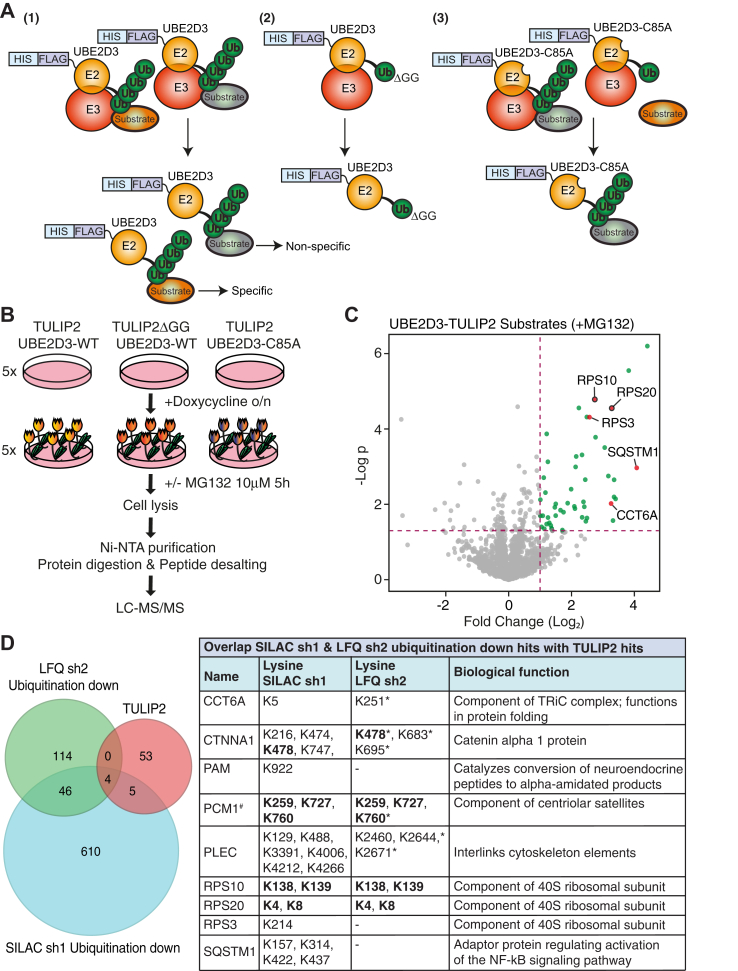
**TULIP2 for identification of direct substrates of UBE2D3.***A*, UBE2D3-TULIP2 rationale. Ubiquitin (Ub) fused to UBE2D3 can be conjugated to a substrate protein following the canonical ubiquitination cascade and pulled down in harsh denaturing conditions together with UBE2D3 and the conjugated substrate (specific and nonspecific substrates in the vicinity (1)). Ub fused to TULIP2-ΔGG cannot be conjugated, thus there will be no conjugated substrates (2). UBE2D3-C85A lacks catalytic activity and is not able to conjugate Ub to its specific targets. However, other E2/E3 enzymes in the surrounding are still able to use the fused Ub for their substrates, named nonspecific substrates (3). *B*, overview experimental approach of TULIP2 methodology. HeLa cells containing UBE2D3-TULIP2, UBE2D3-TULIP2ΔGG, and UBE2D3-C85A-TULIP2 expression cassettes were induced o/n with doxycycline, not treated or treated with proteasome inhibitor (MG132), and lysed. TULIP2 conjugates were Ni–NTA purified, proteins were digested, and peptides were desalted and analyzed by LC–MS/MS. *C*, Volcano plot depicting the UBE2D3 conjugates (n = 4). *Green dots* represent statistically enriched proteins in the UBE2D3-TULIP2 samples compared with UBE2D3-TULIP2ΔGG samples after proteasome inhibition for *p* value = 0.05 and S0 = 0.1. *Red dots* correspond to top hits significantly downregulated in their ubiquitination in SILAC *Ube2d3* sh1 experiments. *Red dots* with a *black stroke* around them also overlap with top hits significantly downregulated in their ubiquitination in LFQ *Ube2d3* sh2 experiments. *D*, Venn diagrams illustrating the overlap between TULIP2 hits (+MG132 and no MG132) and proteins significantly downregulated in their ubiquitination in the SILAC *Ube2d3* sh1 and LFQ *Ube2d3* sh2 diGly proteomics. Table on the *right* shows overlapping hits between TULIP2 and SILAC sh1 and LFQ sh2, including modified lysines and the biological functions of these proteins. *Bold lysines* overlap between SILAC sh1 and LFQ sh2. *Asterisk* indicates that these sites were decreased in their ubiquitination but not significantly (*p* > 0.05). “#” indicates that only lysines that overlap between SILAC sh1 and LFQ sh2 were selected, as a large number of peptides significantly decreased in their ubiquitination were found for PCM1 in SILAC sh1 experiments. LFQ, label-free quantitation; Ni–NTA, nickel–nitrilotriacetic acid; SILAC, stable isotope labeling of amino acids in cell culture; TULIP2, Targets of Ubiquitin Ligases Identified by Proteomics 2.

The diGly proteomics approaches that we used here successfully identified a considerable number of potential novel targets for UBE2D3 but are not able to distinguish between direct and indirect substrates of UBE2D3. Therefore, we used the TULIP2 methodology (21), originally set up to identify substrates of E3 ligases through E3 ligase–ubiquitin fusions, to confirm substrates as direct targets of UBE2D3. TULIP2 methodology makes use of lentiviral doxycycline-inducible expression constructs with an activated ubiquitin C-terminally fused to the E3 ligase of interest, a linker containing a 10× HIS tag in between the E3 ligase and ubiquitin, and a 10× HIS-FLAG tag N-terminal of the E3 ligase. This allows for nickel–NTA purification and the use of harsh buffers to distinguish between substrates and interactors (21). Here, we used TULIP2 for the first time to identify targets of an E2 enzyme (Fig. 5*A*). After generation of HeLa cells with stably integrated TULIP2 constructs with UBE2D3 WT, UBE2D3 C85A (catalytically inactive mutant), and UBE2D3 WT with a ubiquitin lacking the diGly motif (ΔGG) as a negative control, expression was induced with doxycycline and the cells were untreated or treated with proteasome inhibitor (MG132) to prevent degradation of ubiquitinated substrates of UBE2D3. Subsequently, substrates covalently attached to TULIP2-UBE2D3 constructs, through interaction of UBE2D3 with different E3 ligases, were purified using Ni–NTA beads, and peptides were analyzed by MS (Fig. 5, *A* and *B* and supplemental Fig. S5*A*).

The TULIP2 experiments identified 62 targets in total (with and without proteasome inhibitor combined) (supplemental Table S16). TULIP2 confirmed nine potential substrates of UBE2D3 with decreased ubiquitination in the SILAC diGly proteomics as direct targets of UBE2D3 (Fig. 5, *C* and *D* and supplemental Fig. S5*B*). These include not only the top hits RPS10 and RPS20, but also SQSTM1, which is a known target of UBE2D3 (63). In addition, TULIP2 identified PCNA, another previously identified target of UBE2D3 (71), demonstrating that this methodology succeeds in identifying targets of E2 enzymes. Moreover, at least four of these nine targets, PCM1, PLEC, RPS10, and RPS20, were also independently confirmed by the LFQ *Ube2d3* sh2 diGly proteomics (Fig. 5, *C* and *D* and supplemental Fig. S5*B*; supplemental Table S16). The TULIP2 data confirm the top hits from our SILAC sh1 and LFQ sh2 diGly proteomics experiments, RPS10 and RPS20, as direct targets of UBE2D3 and emphasize the importance of UBE2D3 in regulation of mRNA translation, in line with the IPA and STRING results. Therefore, we continued with further validating RPS10 and RPS20.

### RPS10 and RPS20 are Ubiquitinated *in Vivo* by UBE2D3

**Fig. 6:**
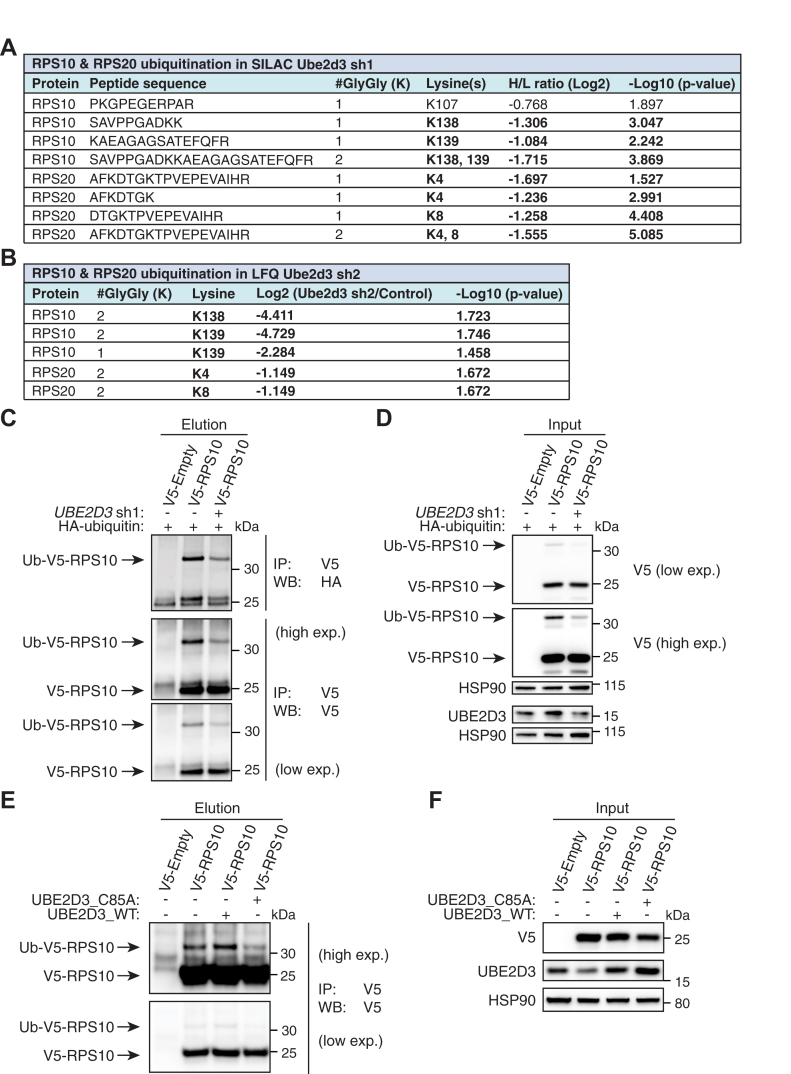
**UBE2D3 regulates ubiquitination of RPS10.***A*, table with significantly changed diGly-modified peptides found for RPS10 and RPS20 in the SILAC-based diGly proteomics, including the number (#) and sites of modified lysines, average H/L normalized log2 ratios, and statistical significance (hits are considered significant when −log10 *p* value ≥ 1.3 or *p* value ≤ 0.05). *Bold lysines* overlap between SILAC sh1 and LFQ sh2. *B*, table with significantly changed diGly-modified peptides found for RPS10 and RPS20 from the LFQ diGly proteomics, including the number (#) and sites of modified lysines, average log2 ratios, and statistical significance (*p* value). *Bold lysines* overlap between SILAC sh1 and LFQ sh2. *C* and *D*, immunoprecipitation (IP) assay in 293T cells ± *UBE2D3* sh1 transfected with V5-tagged RPS10 and hemagglutinin-tagged ubiquitin. Immunoblots representing eluates of the V5-IP, showing decreased ubiquitination of RPS10 in UBE2D3-depleted cells (*C*) and immunoblots of input samples (*D*). Representative blots of n = 3 are shown. *E* and *F*, IP in control 293T cells or overexpressing either UBE2D3 WT or a catalytically inactive mutant of UBE2D3 (C85A), transfected with V5-tagged RPS10. Immunoblots representing eluates of the V5-IP, showing increased ubiquitination of RPS10 in UBE2D3 WT cells and decreased ubiquitination of RPS10 in UBE2D3 C85A cells (*E*) and immunoblots of input samples (*F*). Representative blots of n = 2 are shown. LFQ, label-free quantitation; SILAC, stable isotope labeling of amino acids in cell culture.

Stalling of ribosomes during protein synthesis activates the RQC pathway to eliminate aberrant polypeptides that are potentially deleterious. Stalling of translation can occur for multiple reasons, such as truncated or damaged mRNA, excessive mRNA secondary structure, prematurely polyadenylated mRNA, and insufficient amounts of an amino acid or a tRNA (72). Upon encountering a prematurely polyadenylated mRNA, the ribosome stalls and the E3 ligase ZNF598 ubiquitinates the 40S ribosomal proteins RPS3, RPS3A, RPS10, and RPS20 at lysine 214 (K214), lysine 249 (K249), lysines 138/139 (K138/139), and lysines 4/8 (K4/8), respectively (22, 23, 24). This results in ribosome disassembly and activation of the RQC pathway and subsequent ubiquitination and degradation of the nascent polypeptide (72, 73). ZNF598 was previously shown to interact with UBE2D3, and depletion of UBE2D3 partially impaired RQC, suggesting that UBE2D3 may be involved in ubiquitination of ZNF598 substrates (22). We found significantly reduced RPS3-K214 ubiquitination in the SILAC *Ube2d3* sh1 diGly proteomics but no change in RPS3-K214 ubiquitination in the LFQ *Ube2d3* sh2 diGly proteomics. We found RPS3A-K249 ubiquitination reduced in the LFQ sh2 experiments, but this was not statistically significant and therefore not considered as a hit, whereas RPS3A peptides with Ub modification on K249 were not identified in SILAC sh1 experiments (supplemental Tables S7, S9, S10, and S11). Given the lack of reproducibility in RPS3 and RPS3A modification between *Ube2d3* sh1 and sh2 experiments, we decided to not continue with these proteins. However, from the different diGly-peptides recovered for RPS10, all peptides ubiquitinated on K138 and/or K139 were decreased in UBE2D3-depleted cells, with both shRNAs (Fig. 6, *A* and *B*). Of these, peptides ubiquitinated on both K138 and K139 were more frequently observed than peptides with only one of these sites modified. From the diGly-peptides recovered for RPS20, all peptides ubiquitinated on K4 and/or K8 were decreased in UBE2D3 knockdown cells, with both shRNAs (Fig. 6, *A* and *B*). Also here, mainly peptides modified on both K4 and K8 were found. Importantly, the abundance of RPS10 or RPS20 was not affected by UBE2D3 depletion, thus the observed decrease in their ubiquitination is not caused by lower protein abundance (supplemental Tables S1 and S4). This indicates that RPS10(K138/139) and RPS20(K4/8) ubiquitination by ZNF598 *in vivo* is dependent on UBE2D3.

Ubiquitination of RPS10 on K138/139 is critical for proper RQC (22, 23). To further address the ubiquitination of RPS10 *in vivo* and validate the results from the ubiquitin diGly proteomics and TULIP2 analyses with independent *UBE2D3* shRNAs and cell line, we transfected WT or UBE2D3-depleted HEK 293T cells with V5-tagged RPS10 and HA-tagged ubiquitin and immunoprecipitated V5-RPS10. Indeed, when UBE2D3 was depleted with two independent shRNAs, V5-RPS10 ubiquitination was decreased, indicating that ubiquitination of RPS10 *in vivo* is to a considerable extent dependent on UBE2D3 (Fig. 6, *C* and *D* and supplemental Fig. S6, *A*–*C*). In addition, we also confirmed decreased RPS20 ubiquitination upon UBE2D3 depletion in HEK 293T cells (supplemental Fig. S6, *D* and *E*). Moreover, we show that ubiquitination of RPS10 by UBE2D3 *in vivo* is dependent on the catalytic activity of UBE2D3, as overexpression of UBE2D3 WT, but not UBE2D3 C85A, resulted in increased ubiquitination of RPS10 (Fig. 6, *E* and *F*). Hence, despite redundancy between UBE2D family members and E2 enzymes in general, UBE2D3 seems to be the main E2 that ubiquitinates RPS10 in the MEFs used in our proteomics experiments as well as in the human HeLa cells used in TULIP2 experiments, and in HEK 293T cells used for *in vivo* ubiquitination experiments. Thus, here we demonstrate that UBE2D3 ubiquitinates RPS10 and RPS20 *in vivo*, supporting a role for UBE2D3 in the degradation of aberrant nascent polypeptides through the RQC pathway.

## Discussion

Identification of a complete set of targets of ubiquitin machinery enzymes is challenging. Different methods have been developed in the past to try tackle this problem, such as functional genomics using siRNA libraries, global protein stability profiling, and affinity-based proteomics (14, 74, 75, 76). This has resulted in the identification of targets of different E3 ligases, including HUWE1, PARKIN, SCF^Saf1^ and HRD1, and of DUBs such as USP32 and CYLD (13, 14, 15, 75, 77, 78). For the limited set of E2 enzymes that exist in cells for the ubiquitination of all proteins targeted by E3s, it is even more challenging to identify the probably larger number of targets of a particular E2. Here, we combined quantitative ubiquitinome profiling by SILAC-based or label-free ubiquitin diGly proteomics with depletion of UBE2D3 to identify novel *in vivo* targets of UBE2D3, one of the most promiscuous E2 enzymes *in vitro*. In addition, we used a modified TULIP2 approach to confirm potential substrates of UBE2D3 as direct targets. We show that UBE2D3-depleted cells undergo global proteome and ubiquitinome changes, of which the ubiquitinome changes are most prominent. Our analysis revealed that UBE2D3 affects the abundance and/or ubiquitination of proteins involved in a wide variety of processes, in particular proteins belonging to molecular pathways related to metabolism and mRNA translation. Interestingly, our results indicate that in contrast to the very promiscuous activity of UBE2D3 *in vitro*, UBE2D3 is a rather selective E2 enzyme *in vivo*, affecting approximately 2% of the global proteome and ±10% of the recovered diGly-modified peptides. Although it is possible that some targets or UBE2D3 are not detected in our approaches because of incomplete ablation of UBE2D3 activity or redundant activities of other E2 enzymes.

In our global proteome data, we identified new potential targets of UBE2D3 that function in retinol metabolism and respond to retinol signaling. In particular, both the SILAC-based and label-free proteomics data identified increased abundance of CRABP1 upon UBE2D3 depletion with two independent shRNAs, which we confirmed in Western blots of cell lysates (Fig. 2*G*). Interestingly, ALDH1A1, identified as a hit with decreased abundance in both LFQ experiments with two independent *Ube2d3* shRNAs, is also involved in retinol signaling (Fig. 2, *B*, *C*, and *F*). In the SILAC-based proteomics, ALDH1A1 also decreased in abundance but was not selected as a hit from that data because its change did not meet the threshold for significance (−log10 *p* value = 1.27). ALDH1A1 is a retinol dehydrogenase that converts retinaldehyde to RA and thereby plays an important role in retinol metabolism (79, 80). Moreover, RBP1 (retinol-binding protein 1) was also significantly increased in abundance upon UBE2D3 depletion in the SILAC-based proteomics (supplemental Tables S1 and S2). Although RBP1 peptides were unfortunately not identified in the label-free proteomics, by regulating the abundance of CRABP1, ALDH1A1, and RBP1, UBE2D3 might be an important multilevel regulator of retinol metabolism and downstream responses to retinoids. This is interesting in the light of cancer treatment with ATRA, a derivative of retinol. ATRA regulates gene expression and cell differentiation and is used to treat acute promyelocytic leukemia, in which it induces cell cycle arrest and results in clinical remission. Interestingly, UBE2D3 was found upregulated in ATRA-treated NB4 acute promyelocytic leukemia cells, leading to UBE2D3-mediated degradation of cyclin D1 and cell cycle arrest (11). This hints at a potential feedback mechanism in which UBE2D3, as regulator of retinol metabolism and responses, is itself responding to ATRA-mediated retinoid signaling, perhaps to contribute to retinoid signaling homeostasis. Interestingly, when UBE2D3 is depleted, NB4 cells were found to acquire resistance to ATRA treatment. It is tempting to speculate that this could relate to increased retinol metabolism and retinoid response proteins caused by reduced UBE2D3 activity.

Furthermore, we found significantly decreased protein levels of TSPAN8 in UBE2D3-depleted cells, both in our proteomics data and during validation by Western blotting of cell lysates. Analysis of TSPAN8 transcript levels indicate that this decrease is due to regulation of TSPAN8 at the protein level. The mechanism underlying this regulation by UBE2D3, whether it involves regulation of degradation and/or of translation, and what functional consequences it has, are all unclear, but could be interesting directions for further research. TSPAN8 is overexpressed (on protein and/or mRNA level) in various cancers, including pancreatic, lung, breast, ovarian, colon, liver, and gastric cancers (40). High TSPAN8 levels in tumors promote proliferation, migration, angiogenesis, and metastasis, and correlate with poor survival. On the other hand, TSPAN8 depletion was shown to reduce cell viability, proliferation and invasion of different tumors and increase apoptosis (40). It would be interesting to address whether this is regulated by UBE2D3 and whether UBE2D3 levels are potentially also increased in these cancers. Interestingly, a potential mechanism through which UBE2D3 might affect TSPAN8 levels is suggested by our observation that UBE2D3 depletion results in increased ubiquitination of 14-3-3θ on lysine 9 (K9), both in SILAC sh1 as well as LFQ sh2 experiments. 14-3-3 proteins are adaptor and chaperone proteins that function in many different processes such as signal transduction, protein trafficking, and apoptosis (58, 81). Interestingly, 14-3-3θ was recently reported to promote TSPAN8 translocation into the nucleus in MDA-MB-231 cells (human breast adenocarcinoma) (82). Although the consequences of TSPAN8 translocation to the nucleus are as yet unexplored, it could potentially rescue TSPAN8 from being degraded in the cytoplasm. An interesting direction for further research would be to address whether increased ubiquitination of 14-3-3θ in UBE2D3-depleted cells changes the activity or localization of 14-3-3θ and thereby interferes with 14-3-3θ-mediated TSPAN8 translocation to the nucleus, and thereby its potential protection from degradation in the cytoplasm.

We particularly focused on the role of UBE2D3 in the RQC pathway, a pathway that acts upon ribosome stalling, including at prematurely polyadenylated mRNAs, and senses 60S ribosomal subunits obstructed with peptidyl-tRNA (72, 73). RQC is associated with multiple layers of ubiquitin-mediated control. These include ubiquitination of the 40S ribosomal protein RPS10 at K107 by the E3 ligase MKRN1 at the start of poly(A) sequences to stall the ribosome (83), followed by ubiquitination of RPS10 at K138/139 and RPS20 at K4/8 by the E3 ligase ZNF598, which recognizes collision of the next ribosome with the stalled ribosome (22, 23, 24, 84). Subsequently, RACK1 facilitates RPS2 and RPS3 ubiquitination (24, 85), the ribosome disassembles and the RQC pathway is activated, and the nascent polypeptide is polyubiquitinated by the E3 ligase listerin (LTN1). This is followed by recruitment of the AAA-ATPase VCP that extracts the ubiquitinated peptide, which is then degraded by the proteasome (72, 73). Here, we found that ubiquitination of the 40S ribosomal proteins RPS10 and RPS20, on respectively, lysines K138/139 and K4/8, is governed by UBE2D3 *in vivo*, and through direct immunoprecipitation of RPS10 and RPS20 from cells, we confirmed that their ubiquitination is indeed dependent on UBE2D3. In addition, through TULIP2 methodology, we identified RPS10 and RPS20 as direct substrates of UBE2D3. Moreover, immunoprecipitation experiments in HEK 293T cells confirmed that the catalytic activity of UBE2D3 as an E2 enzyme is required for ubiquitination of RPS10 *in vivo*, further supporting that RPS10 is a direct target of UBE2D3. In the absence of ZNF598-mediated ubiquitination of RPS10-K138/139 and RPS20-K4/8, execution of RQC is compromised (22, 23, 24). Garzia *et al.* (22) demonstrated before that depletion of UBE2D3 partially impairs RQC and that ZNF598 and UBE2D3 interact with each other, which points at UBE2D3 as the potential candidate E2 enzyme that functions together with ZNF598 in the ubiquitination of RPS10 and RPS20. Our findings confirm that UBE2D3 is indeed the E2 that is required for the ubiquitination of these residues by ZNF598 *in vivo*. UBE2D1, a closely related and partially redundant family member of UBE2D3, was shown to be able to act *in vitro* with ZNF598 in the ubiquitination of RPS3A, RPS10, and RPS20 (23). However, our results showing that RPS10/20 ubiquitination significantly drops in cells with impaired UBE2D3 activity and that RPS10 and RPS20 are direct targets of UBE2D3, suggest that, although UBE2D1 may contribute, UBE2D3 is the main E2 enzyme ubiquitinating these proteins *in vivo*, in the mouse and human cell lines we used.

Our ubiquitin diGly proteomics data also showed decreased ubiquitination of RPS2, RPS3, and RPS3A at the previously reported ubiquitinated lysines (K275 for RPS2, K214 for RPS3, and K249 for RPS3A) in UBE2D3-depleted cells (supplemental Tables S9 and S10). While this suggests RPS2 and RPS3 as potential targets of UBE2D3, it could also reflect an indirect consequence of the reduced RPS10 ubiquitination in UBE2D3-depleted cells, as defective RPS10 ubiquitination was found associated with partially decreased RPS2 and RPS3 ubiquitination (85). UBE2D3 depletion only slightly (±1.7-fold change) affects K107 ubiquitination on RPS10 (Fig. 6*A* and supplemental Table S9), suggesting that UBE2D3 does not operate at the level of MKRN1, preceding RQC and prior to ZNF598 activity. However, besides RPS10 and RPS20 and the effects on RPS2, RPS3, and RPS3A, we also observed impaired ubiquitination of other, later, components of the RQC pathway when UBE2D3 was depleted. Both listerin (LTN1) and VCP showed reduced ubiquitination at multiple lysines throughout these proteins in both SILAC sh1 and LFQ sh2 datasets. However these modifications only reached statistical significance in the SILAC sh1 experiments, and their functional importance is thus far unclear (supplemental Tables S9 and S10). Nevertheless, while these results clearly implicate UBE2D3 in RQC at the level of RPS10 and RPS20 ubiquitination by ZNF598, UBE2D3 might have a more widespread role in RQC by also affecting the ubiquitination of other proteins acting in this pathway.

As RQC is one of the main mechanisms preventing the production and accumulation of defective proteins (86, 87), by functioning in this pathway, UBE2D3 could have an important role in preserving cellular functions and in preventing diseases linked to protein aggregate formation. Moreover, UBE2D3 also functions in the PQC pathway that targets misfolded proteins for proteasomal degradation (88) and as we observed in our own data, UBE2D3 might also regulate autophagic PQC *via* SQSTM1, HSPA5, and TAX1BP1. This indicates that next to preventing the production of defective proteins *via* RQC, UBE2D3 can also contribute to the removal of dysfunctional proteins and their aggregates *via* PQC. SQSTM1 and HSPA5 dysregulation has been implicated in neurodegenerative disorders, such as Alzheimer’s disease and Parkinson’s disease (89, 90). By regulating SQSTM1 and HSPA5 ubiquitination and regulating SQSTM1 activity, UBE2D3 could potentially contribute to clearance of aggregates and thereby help in reducing the chance of development of neurodegenerative diseases. Interesting to note, as it supports a potential role for UBE2D3 in pathology related to its functions in RQC and (autophagic) PQC, the expression of UBE2D3 was found decreased at early stages of Parkinson’s disease in mouse brains (91).

Altogether, by combining UBE2D3 depletion with SILAC-based and label-free quantitative ubiquitin diGly proteomics, we successfully identified proteins whose ubiquitination and/or abundance *in vivo* is affected by UBE2D3. This gives novel insights into the spectrum of potential *in vivo* UBE2D3 targets and exposes important *in vivo* roles for UBE2D3, in particular in retinol metabolism and signaling, RQC, and autophagic PQC. We established multiple new links, and for some roles, where indications already existed from previous work that UBE2D3 might be affecting key factors in these pathways, we were able to confirm such links *in vivo* and demonstrate how UBE2D3 changes the levels or ubiquitination of these factors. Most notably, we found that UBE2D3 is the primary E2 enzyme that ubiquitinates RPS10 and RPS20 *in vivo*. It will be interesting to further explore how UBE2D3 affects different cellular processes and pathologies in which retinol metabolism, RQC and PQC, play an important role and which E3 enzymes act together with UBE2D3 to ubiquitinate proteins involved in these processes.

## Data Availability

### SILAC-Based and LFQ diGly Proteomics

MS raw data and data for protein identification and quantification were submitted as supplemental tables to the ProteomeXchange Consortium *via* the PRIDE partner repository (92) with the data identifier PXD035045. For reviewing purposes, the following credentials can be used:

Username: reviewer_pxd035045@ebi.ac.uk

Password: IRIBP4Jh

### TULIP2

The MS proteomics data have been deposited to the ProteomeXchange Consortium *via* the PRIDE partner repository with the dataset identifier PXD026054. For reviewing purposes, the following credentials can be used:

Username: reviewer_pxd026054@ebi.ac.uk

Password: GMBhTQpr

### For LFQ Proteomics, Annotated Spectra Have Been Deposited to MS-Viewer

For proteome, the link is: https://msviewer.ucsf.edu/prospector/cgi-bin/mssearch.cgi?report_title=MS-Viewer&search_key=3opswum57r&search_name=msviewer (key: 3opswum57r), and for the diGly dataset, the link is: https://msviewer.ucsf.edu/cgi-bin/mssearch.cgi?report_title=MS-Viewer&search_key=kapdokzmqp&search_name=msviewer (key: kapdokzmqp).

For TULIP2, MS annotated spectra have been deposited to MS-Viewer: https://msviewer.ucsf.edu/cgi-bin/mssearch.cgi?report_title=MS-Viewer&search_key=lqf88o5m68&search_name=msviewer (key: lqf88o5m68).

## Supplemental data

This article contains supplemental data.

## Conflict of interest

The authors declare no competing interests.

## Supplemental Data

Supplemental Figure S1 **Validation of UBE2D3 depletion in proteomics experiments and numbers of identified proteins and peptides in SILAC *Ube2d3* sh****1 experiments.***A*, Representative immunoblots for UBE2D3 depletion in the SILAC-based (diGly) proteomics experiments. *B*, Immunoblots for UBE2D3 depletion in all three replicates of the LFQ (diGly) proteomics. *C*, Table with numbers of identified proteins in all six SILAC *Ube2d3* sh1 replicates, including numbers of proteins that are ≥1.5 fold increased or decreased in abundance upon UBE2D3 depletion. *D*, Table with identified diGly peptides and the effect of UBE2D3 depletion on the ubiquitination status of diGly modified peptides in all SILAC *Ube2d3* sh1 experiments. *Asterisk* indicates that these numbers are based on a cut-off of ≥1.5-fold increase or decrease in ubiquitination (increase: log2 ≥ 0.585; decrease: log2 ≤ −0.585).

Supplemental Figure S2 **Validation of CRABP1 and TSPAN8 as targets of UBE2D3.***A*, Two additional biological replicates for CRABP1 and TSPAN8 protein levels in *Ube2d3* sh1 cells and three additional biological replicates for CRABP1 and TSPAN8 protein levels in *Ube2d3* sh2 cells are shown for the immunoblot experiments in Figure 2*G*. *B*, Quantification of CRABP1 protein levels relative to control (scrambled) shRNA cells in DMSO. Quantified from immunoblots shown in Figure 2*G* and supplemental Fig. S2*A*. The mean ± SD is shown and each *dot* represents an independent experiment. For *Ube2d3* sh1: n = 3 at 4 h and n = 2 at 16 h of DMSO or MG132 treatment. For *Ube2d3* sh2: n = 4 at 4 h, n = 1 at 8 h and n = 4 at 16 h of DMSO or MG132 treatment. Statistical significance was calculated using a two-tailed Student *t*-test. Differences in CRABP1 protein levels between control shRNA and *Ube2d3* shRNA samples did not reach statistical significance. *C* and *D*, q-RT PCR analysis of *Crabp1* (*C*) and *Tspan8* (*D*) mRNA levels in MEFs. The mean ± SD for three independent experiments is shown, except for *Crabp1* expression in *Ube2d3* sh2, which represents an n = 2. Each *dot* represents an independent experiment. Statistical significance was calculated using a two-tailed Student *t*-test (ns, non-significant (*p* ≥ 0.05), ∗*p* = 0.0303).

Supplemental Figure S3 **Venn diagrams of proteins increased in ubiquitination upon UBE2D3 depletion.***A*, Venn diagrams illustrating the overlap between known UBE2D3 interactors (*BioGRID*, *IntAct*, *MINT*, *STRING* and *UniProt* databases) and proteins that go up in ubiquitination upon UBE2D3 depletion in the SILAC diGly proteomics experiments. The table below shows the overlapping hits and their biological function. *B*, Venn diagrams illustrating the overlap between known UBE2D3 interactors and proteins that go up in ubiquitination upon UBE2D3 depletion in the LFQ sh2 experiments. The table below shows the overlapping hits and their biological function.

Supplemental Figure S4 **Decreased protein abundance and ubiquitination upon UBE2D3 depletion.***A*, STRING analysis of proteins that are decreased in their ubiquitination upon UBE2D3 depletion with sh1 and sh2, zoomed in on a network of proteins involved in mRNA translation. *Lines* between proteins represent known interactions, *dotted line* represent a putative interaction. *B*, Venn diagrams illustrating the overlap between the 50× overlapping hits from the SILAC *Ube2d3* sh1 and LFQ *Ube2d3* sh2 that are significantly downregulated in their ubiquitination and the proteins significantly downregulated in their abundance in the SILAC *Ube2d3* sh1 and LFQ *Ube2d3* sh2 proteomics. *C*, Table showing the overlapping hits in *B*.

Supplemental Figure S5 **UBE2D3-TULIP2 Western blot validation and volcano plot of results without proteasome inhibition.***A*, Expression of UBE2D3-TULIP2, UBE2D3-TULIP2GG and UBE2D3-C85A-TULIP2 in HeLa cells was induced o/n with doxycycline, not treated or treated with proteasome inhibitor (no MG132 or + MG132) and lysed. TULIP2 conjugates were purified following TULIP2 methodology. 0.1% of whole cell extract serves as input and 5% of the purified proteins serves as pull downs (PDs). Ponceau S is provided as a loading control. *B*, Volcano plot showing UBE2D3 substrates from TULIP2 experiments (n = 4) without proteasome inhibition (no MG132). *Green**dots* indicate statistically enriched proteins in the UBE2D3-TULIP2 samples compared to UBE2D3-TULIP2GG samples for *p*-value = 0.05 and S0 = 0.1. *Red dots* indicate top hits significantly decreased in their ubiquitination in SILAC *Ube2d3* sh1 experiments. *Red dots* with a *black stroke* around them also overlap with top hits significantly decreased in their ubiquitination in LFQ *Ube2d3* sh2 experiments.

Supplemental Figure S6 **RPS10 and RPS20 diGly modified peptides in LFQ sh2, V5-RPS10 expression and IPs for RPS10 and RPS20.***A*, Immunoblotting to verify expression of V5-RPS10 in HEK 293T cells. *B* and *C*, IP assay in 293T cells +/− *UBE2D3* sh2 transfected with V5-tagged RPS10 and HA-tagged ubiquitin. Immunoblots representing eluates of the V5-IP, showing decreased ubiquitination of RPS10 in UBE2D3-depleted cells (*B*) and immunoblots of input samples (*C*). Representative blots of n = 2 are shown. *D* and *E*, Endogenous RPS20 IP assay in 293T cells +/− *UBE2D3* sh2, transfected with HA-tagged ubiquitin. Immunoblots representing eluates of the IgG negative control and RPS20 IPs, showing decreased ubiquitination of RPS20 in UBE2D3-depleted cells (*D*) and immunoblots of input samples (*E*). Representative blots of n = 2 are shown.
