# Supplementary material for: Ubiquitinome Profiling Reveals in Vivo UBE2D3 Targets and Implicates UBE2D3 in Protein Quality Control
Source: Mol Cell Proteomics. 2023 Apr 13;22(6):100548. doi: 10.1016/j.mcpro.2023.100548 (PMC10209342; doi:10.1016/j.mcpro.2023.100548)
Supplement: Supplemental Figure S1 — Validation of UBE2D3 depletion in proteomics experiments and numbers of identified proteins and peptides in SILAC Ube2d3 sh1 experiments.A, Representative immunoblots for UBE2D3 depletion in the SILAC-based (diGly) proteomics experiments. B, Immunoblots for UBE2D3 depletion in all three replicates of the LFQ (diGly) proteomics. C, Table with numbers of identified proteins in all six SILAC Ube2d3 sh1 replicates, including numbers of proteins that are ≥1.5 fold increased or decreased in abundance upon UBE2D3 depletion. D, Table with identified diGly peptides and the effect of UBE2D3 depletion on the ubiquitination status of diGly modified peptides in all SILAC Ube2d3 sh1 experiments. Asterisk indicates that these numbers are based on a cut-off of ≥1.5-fold increase or decrease in ubiquitination (increase: log2 ≥ 0.585; decrease: log2 ≤ −0.585). [file mmc1.pdf]

A

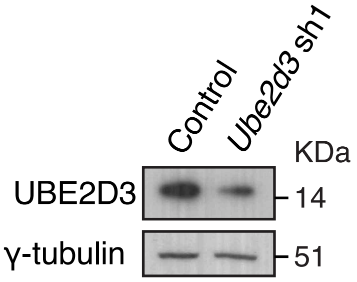

B

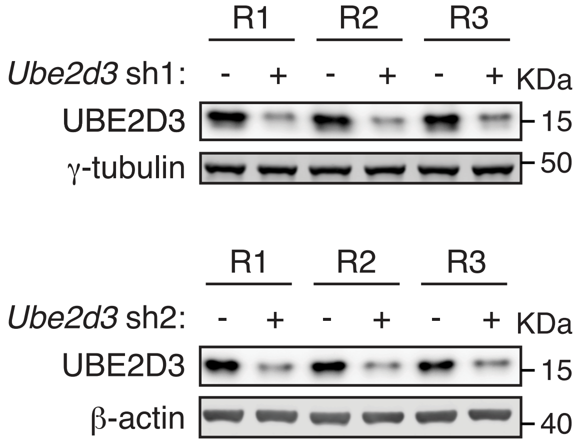

C

Numbers of identified proteins SILAC Ube2d3 sh1			
**Experiment**	**Total # proteins**	**Total Up**	**Total Down**
1	6377	116	209
2	6975	119	152
3	6337	138	206
4	6503	127	232
5	7136	132	199
6	6877	165	176

D

Identified diGly peptides and the effect of *Ube2d3* depletion (SILAC sh1) on their ubiquitination status	
	**# diGly peptides**
Total number of peptides	19599
- with 1 diGly site	18794
- with 2 diGly sites	788
- with 3 diGly sites	16
- with 4 diGly sites	1
Total number with increased ubiquitination*	482
Total number with decreased ubiquitination*	3573
