# Supplementary figures and images for: Ubiquitinome Profiling Reveals in Vivo UBE2D3 Targets and Implicates UBE2D3 in Protein Quality Control
Source: Mol Cell Proteomics. 2023 Apr 13;22(6):100548. doi: 10.1016/j.mcpro.2023.100548 (PMC10209342; doi:10.1016/j.mcpro.2023.100548)

**A**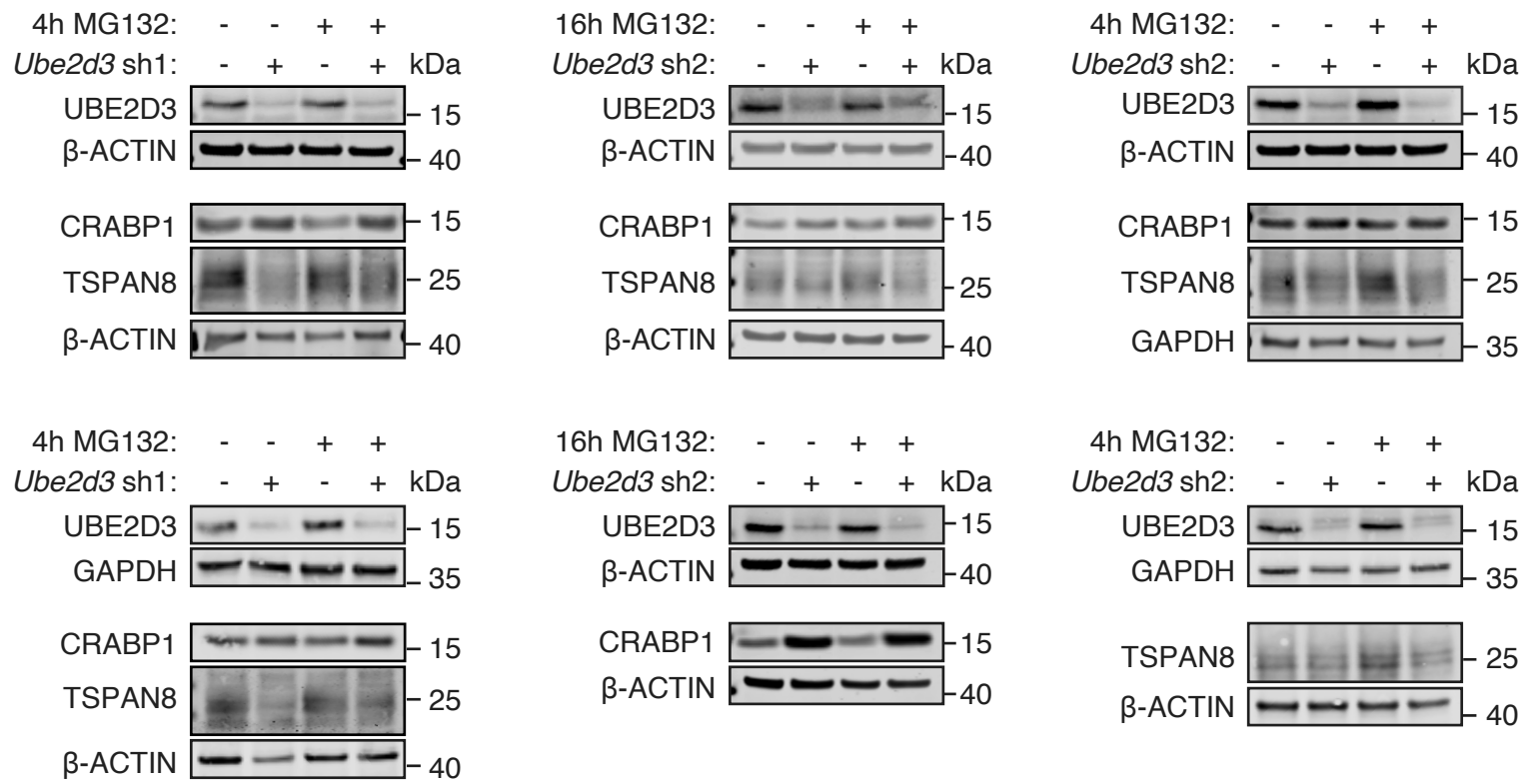**B**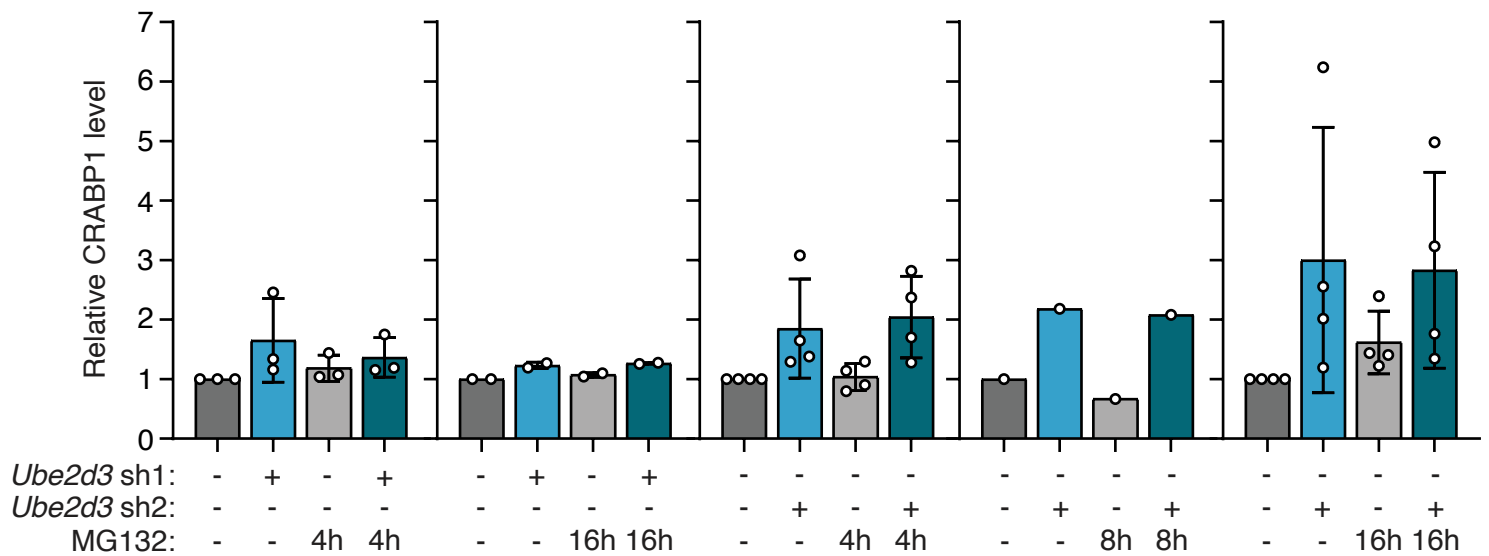**C**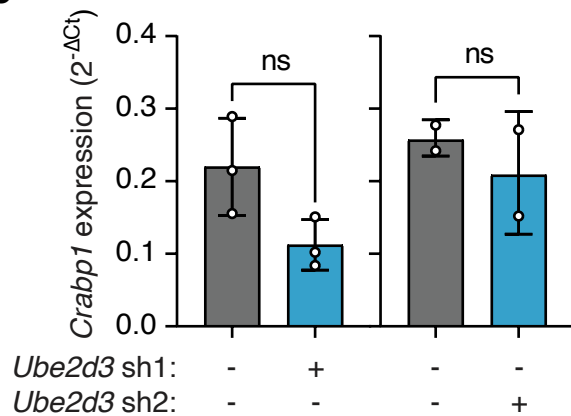**D**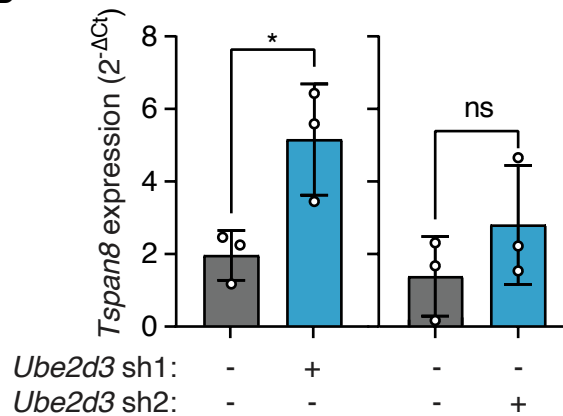

Supplement: Supplemental Figure S2 — Validation of CRABP1 and TSPAN8 as targets of UBE2D3.A, Two additional biological replicates for CRABP1 and TSPAN8 protein levels in Ube2d3 sh1 cells and three additional biological replicates for CRABP1 and TSPAN8 protein levels in Ube2d3 sh2 cells are shown for the immunoblot experiments in Figure 2G. B, Quantification of CRABP1 protein levels relative to control (scrambled) shRNA cells in DMSO. Quantified from immunoblots shown in Figure 2G and supplemental Fig. S2A. The mean ± SD is shown and each dot represents an independent experiment. For Ube2d3 sh1: n = 3 at 4 h and n = 2 at 16 h of DMSO or MG132 treatment. For Ube2d3 sh2: n = 4 at 4 h, n = 1 at 8 h and n = 4 at 16 h of DMSO or MG132 treatment. Statistical significance was calculated using a two-tailed Student t-test. Differences in CRABP1 protein levels between control shRNA and Ube2d3 shRNA samples did not reach statistical significance. C and D, q-RT PCR analysis of Crabp1 (C) and Tspan8 (D) mRNA levels in MEFs. The mean ± SD for three independent experiments is shown, except for Crabp1 expression in Ube2d3 sh2, which represents an n = 2. Each dot represents an independent experiment. Statistical significance was calculated using a two-tailed Student t-test (ns, non-significant (p ≥ 0.05), ∗p = 0.0303). [file mmc2.pdf]

**A**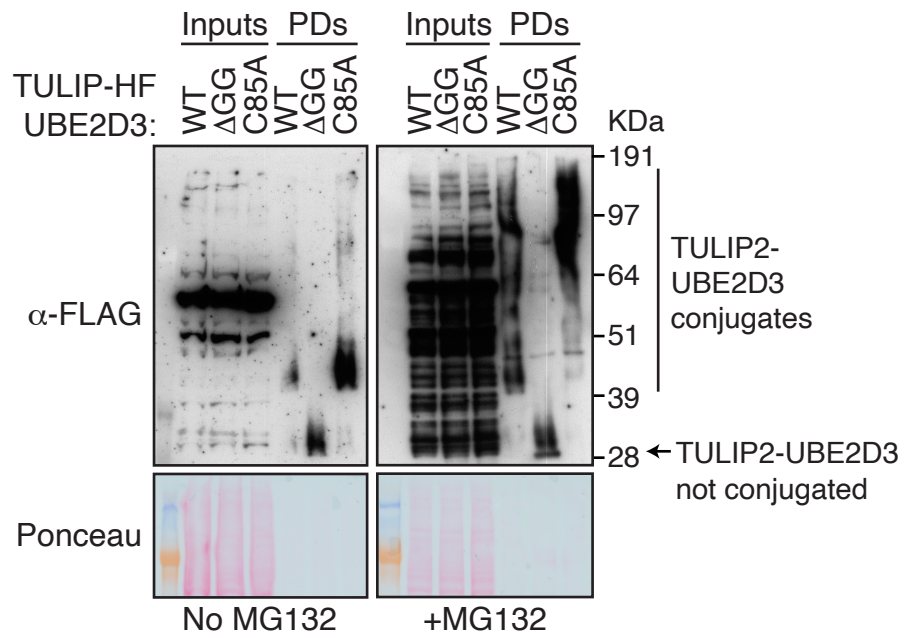**B**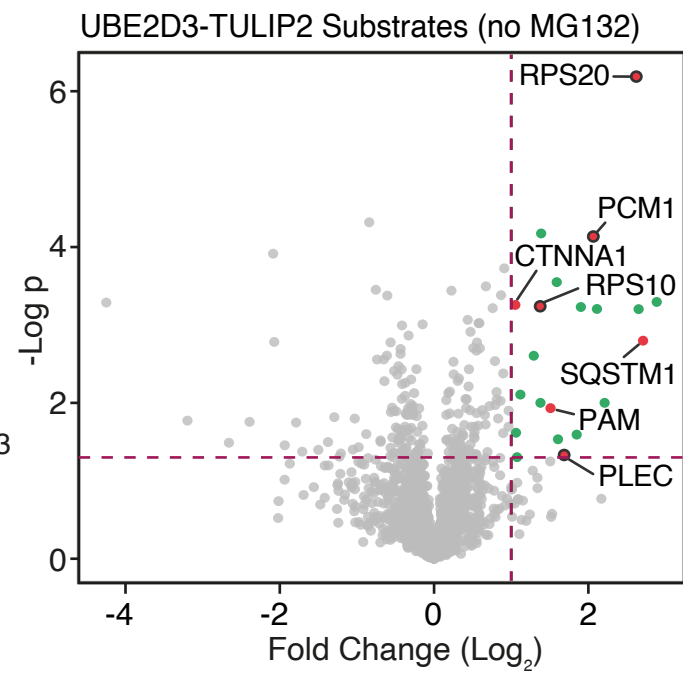

Supplement: Supplemental Figure S5 — UBE2D3-TULIP2 Western blot validation and volcano plot of results without proteasome inhibition.A, Expression of UBE2D3-TULIP2, UBE2D3-TULIP2GG and UBE2D3-C85A-TULIP2 in HeLa cells was induced o/n with doxycycline, not treated or treated with proteasome inhibitor (no MG132 or + MG132) and lysed. TULIP2 conjugates were purified following TULIP2 methodology. 0.1% of whole cell extract serves as input and 5% of the purified proteins serves as pull downs (PDs). Ponceau S is provided as a loading control. B, Volcano plot showing UBE2D3 substrates from TULIP2 experiments (n = 4) without proteasome inhibition (no MG132). Greendots indicate statistically enriched proteins in the UBE2D3-TULIP2 samples compared to UBE2D3-TULIP2GG samples for p-value = 0.05 and S0 = 0.1. Red dots indicate top hits significantly decreased in their ubiquitination in SILAC Ube2d3 sh1 experiments. Red dots with a black stroke around them also overlap with top hits significantly decreased in their ubiquitination in LFQ Ube2d3 sh2 experiments. [file mmc5.pdf]

**A**

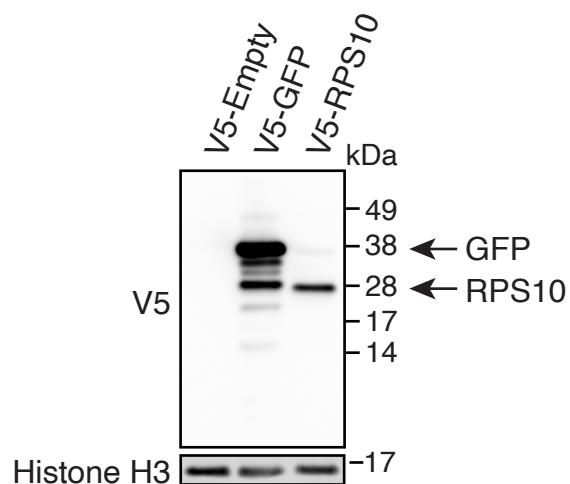

**B**

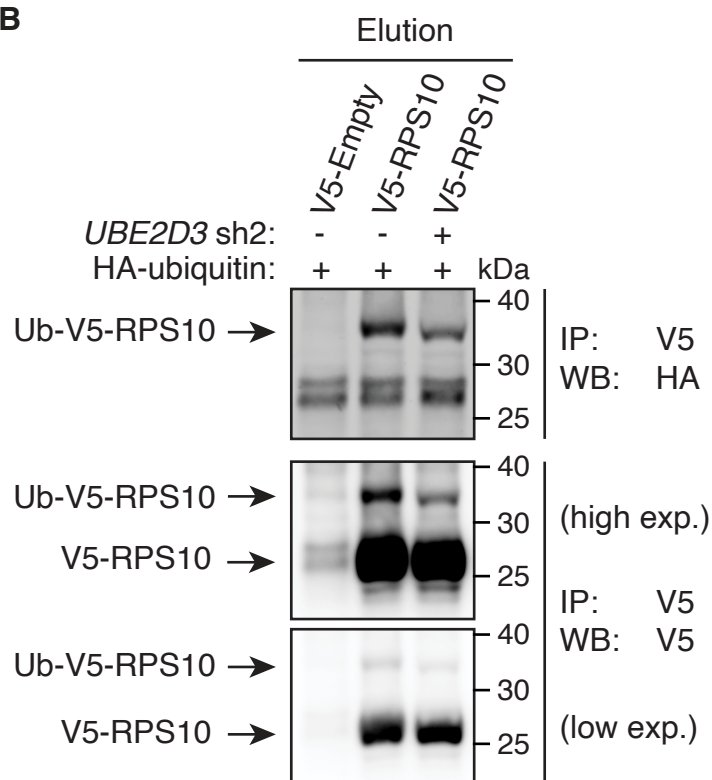

**C**

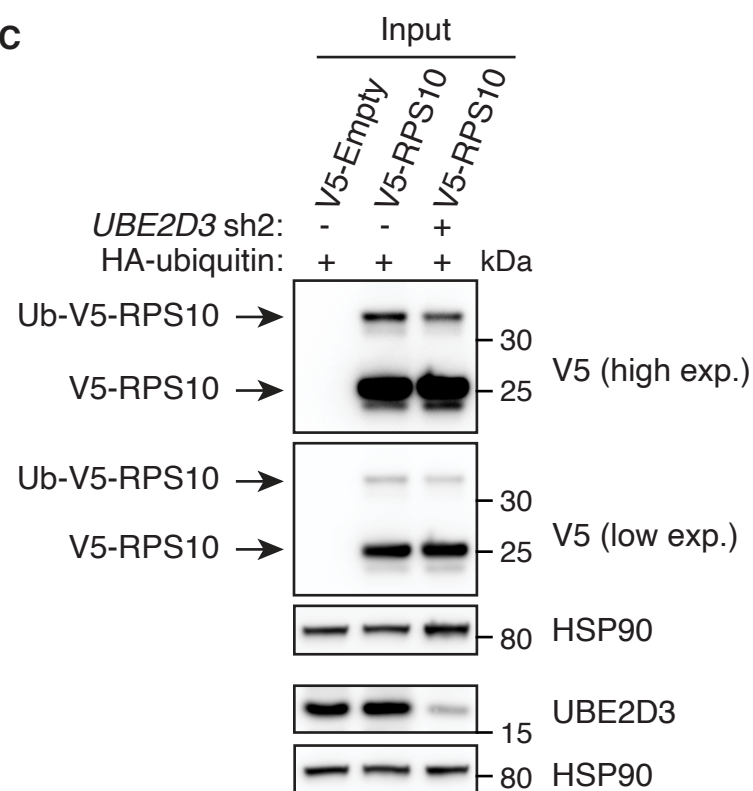

**D**

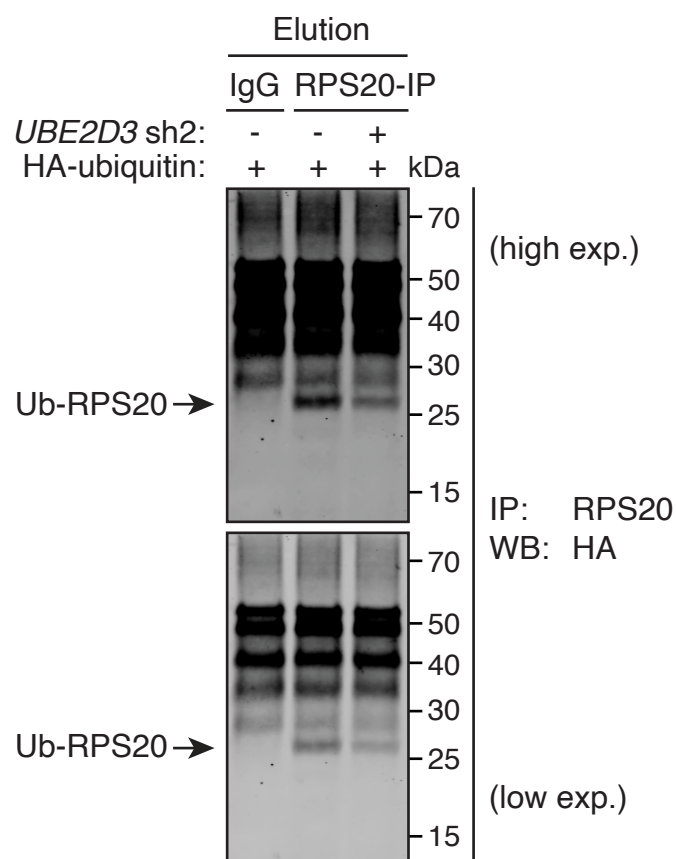

**E**

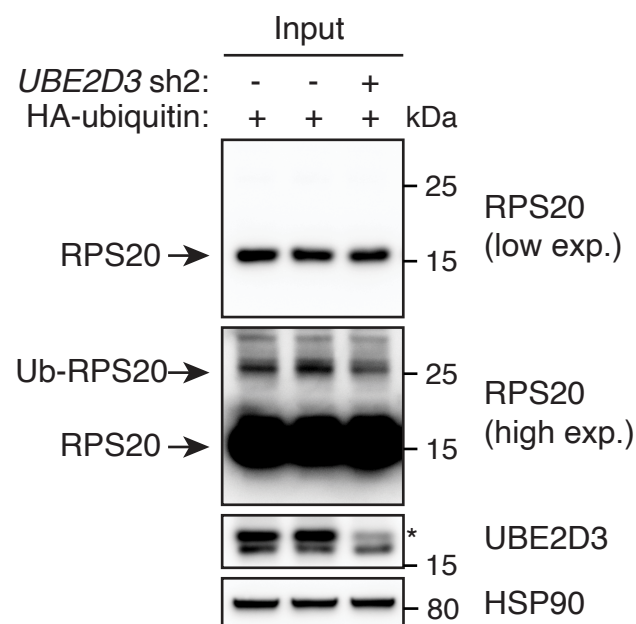

Supplement: Supplemental Figure S6 — RPS10 and RPS20 diGly modified peptides in LFQ sh2, V5-RPS10 expression and IPs for RPS10 and RPS20.A, Immunoblotting to verify expression of V5-RPS10 in HEK 293T cells. B and C, IP assay in 293T cells +/− UBE2D3 sh2 transfected with V5-tagged RPS10 and HA-tagged ubiquitin. Immunoblots representing eluates of the V5-IP, showing decreased ubiquitination of RPS10 in UBE2D3-depleted cells (B) and immunoblots of input samples (C). Representative blots of n = 2 are shown. D and E, Endogenous RPS20 IP assay in 293T cells +/− UBE2D3 sh2, transfected with HA-tagged ubiquitin. Immunoblots representing eluates of the IgG negative control and RPS20 IPs, showing decreased ubiquitination of RPS20 in UBE2D3-depleted cells (D) and immunoblots of input samples (E). Representative blots of n = 2 are shown. [file mmc6.pdf]
