# Supplementary material for: Ubiquitinome Profiling Reveals in Vivo UBE2D3 Targets and Implicates UBE2D3 in Protein Quality Control
Source: Mol Cell Proteomics. 2023 Apr 13;22(6):100548. doi: 10.1016/j.mcpro.2023.100548 (PMC10209342; doi:10.1016/j.mcpro.2023.100548)
Supplement: Supplemental Figure S3 — Venn diagrams of proteins increased in ubiquitination upon UBE2D3 depletion.A, Venn diagrams illustrating the overlap between known UBE2D3 interactors (BioGRID, IntAct, MINT, STRING and UniProt databases) and proteins that go up in ubiquitination upon UBE2D3 depletion in the SILAC diGly proteomics experiments. The table below shows the overlapping hits and their biological function. B, Venn diagrams illustrating the overlap between known UBE2D3 interactors and proteins that go up in ubiquitination upon UBE2D3 depletion in the LFQ sh2 experiments. The table below shows the overlapping hits and their biological function. [file mmc3.pdf]

A

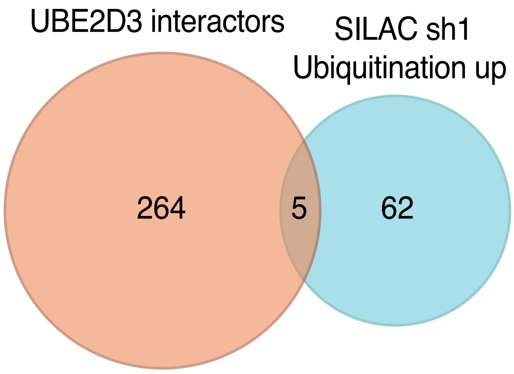

Overlapping hits	
**Name**	**Biological function**
ARIH1	E3 ligase
DZIP3	E3 ligase
FBXW11	F-box protein
UBE2S	E2 ubiquitin-conjugating enzyme
UHRF1	E3 ligase

B

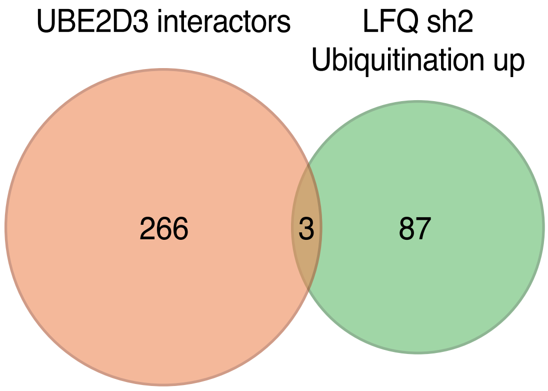

Overlapping hits	
**Name**	**Biological function**
HERC2	E3 ligase
UBE4B	E3 ligase
UBTD1	Ubiquitin domain-containing protein
