# Supplementary material for: Ubiquitinome Profiling Reveals in Vivo UBE2D3 Targets and Implicates UBE2D3 in Protein Quality Control
Source: Mol Cell Proteomics. 2023 Apr 13;22(6):100548. doi: 10.1016/j.mcpro.2023.100548 (PMC10209342; doi:10.1016/j.mcpro.2023.100548)
Supplement: Supplemental Figure S4 — Decreased protein abundance and ubiquitination upon UBE2D3 depletion.A, STRING analysis of proteins that are decreased in their ubiquitination upon UBE2D3 depletion with sh1 and sh2, zoomed in on a network of proteins involved in mRNA translation. Lines between proteins represent known interactions, dotted line represent a putative interaction. B, Venn diagrams illustrating the overlap between the 50× overlapping hits from the SILAC Ube2d3 sh1 and LFQ Ube2d3 sh2 that are significantly downregulated in their ubiquitination and the proteins significantly downregulated in their abundance in the SILAC Ube2d3 sh1 and LFQ Ube2d3 sh2 proteomics. C, Table showing the overlapping hits in B. [file mmc4.pdf]

A

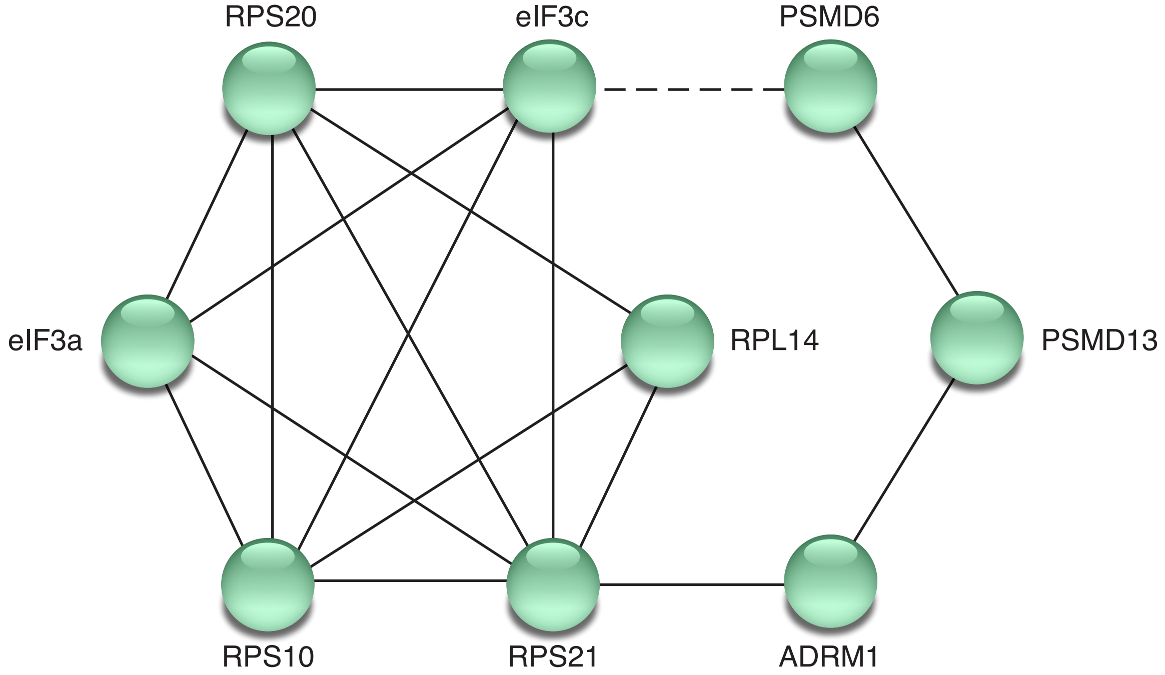

B

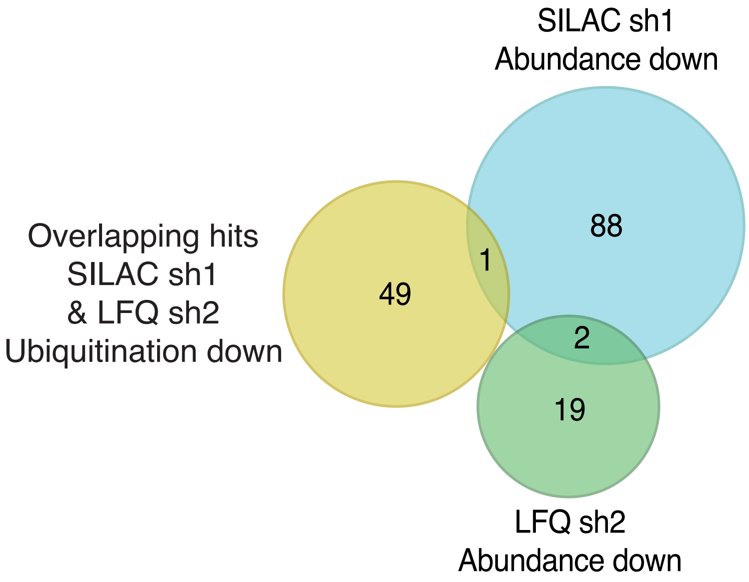

C

Overlapping hits
**Overlapping hits sh1 & sh2 ubiquitination down vs SILAC sh1 abundance down**
UBE2D3
**SILAC sh1 abundance down vs LFQ sh2 abundance down**
TBC1D2<br>TSPAN8
